# Benzothiazole Derivatives Endowed with Antiproliferative Activity in Paraganglioma and Pancreatic Cancer Cells: Structure–Activity Relationship Studies and Target Prediction Analysis

**DOI:** 10.3390/ph15080937

**Published:** 2022-07-28

**Authors:** Rosa Amoroso, Laura De Lellis, Rosalba Florio, Nazaret Moreno, Mariangela Agamennone, Barbara De Filippis, Letizia Giampietro, Cristina Maccallini, Inmaculada Fernández, Rocío Recio, Alessandro Cama, Marialuigia Fantacuzzi, Alessandra Ammazzalorso

**Affiliations:** 1 Department of Pharmacy, “G. d’Annunzio” University of Chieti-Pescara, Via Dei Vestini 31, 66100 Chieti, Italy; rosa.amoroso@unich.it (R.A.); laura.delellis@unich.it (L.D.L.); rosalba.florio@unich.it (R.F.); mariangela.agamennone@unich.it (M.A.); barbara.defilippis@unich.it (B.D.F.); letizia.giampietro@unich.it (L.G.); cristina.maccallini@unich.it (C.M.); alessandro.cama@unich.it (A.C.); 2 Center for Advanced Studies and Technology CAST, Via Luigi Polacchi 11, 66100 Chieti, Italy; 3 Departamento de Química Orgánica y Farmacéutica, Facultad de Farmacia, Universidad de Sevilla, C/Profesor García González, 2, 41012 Sevilla, Spain; nmoreno4@us.es (N.M.); inmaff@us.es (I.F.); rrecioj@us.es (R.R.)

**Keywords:** benzothiazole, antiproliferative, pancreatic cancer, paraganglioma, gemcitabine combination, target prediction

## Abstract

The antiproliferative effects played by benzothiazoles in different cancers have aroused the interest for these molecules as promising antitumor agents. In this work, a library of phenylacetamide derivatives containing the benzothiazole nucleus was synthesized and compounds were tested for their antiproliferative activity in paraganglioma and pancreatic cancer cell lines. The novel synthesized compounds induced a marked viability reduction at low micromolar concentrations both in paraganglioma and pancreatic cancer cells. Derivative **4l** showed a greater antiproliferative effect and higher selectivity index against cancer cells, as compared to other compounds. Notably, combinations of derivative **4l** with gemcitabine at low concentrations induced enhanced and synergistic effects on pancreatic cancer cell viability, thus supporting the relevance of compound **4l** in the perspective of clinical translation. A target prediction analysis was also carried out on **4l** by using multiple computational tools, identifying cannabinoid receptors and sentrin-specific proteases as putative targets contributing to the observed antiproliferative activity.

## 1. Introduction

**Figure 1:**
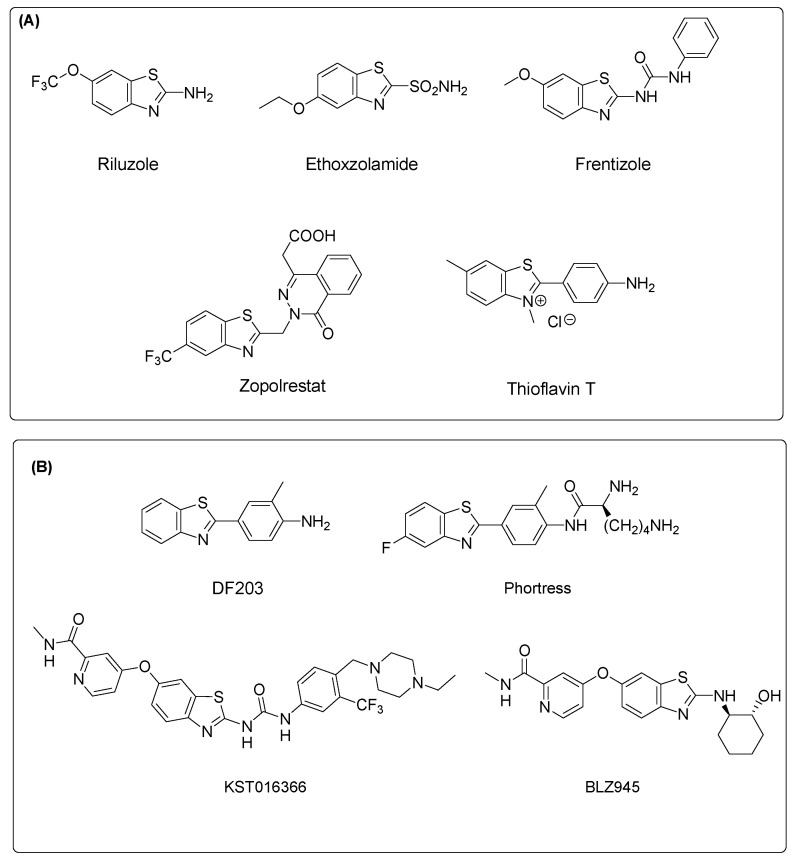
(**A**) Selected marketed drugs containing the benzothiazole nucleus; (**B**) Chemical structures of some benzothiazole derivatives endowed with strong antiproliferative effects in cancer cell models.

Heterocycles represent precious scaffolds in natural and synthetic molecules, endowed with a great variety of biological activities. Benzothiazoles are members of the bicyclic heteroaromatic family, and they are widely used in medicinal chemistry as the scaffolds of several drugs, including antimicrobial, anti-inflammatory, anticonvulsant, neuroprotective and many others [1,2]. The easy functionalization of the aromatic ring of benzothiazole and the 2-amino or 2-mercapto substituents, which are frequently used as building blocks, makes them attractive and reactive components that are useful in organic and medicinal chemistry programs [3,4]. The development of benzothiazole-based drugs has led to a number of derivatives currently marketed to treat different pathologies, such as the neuroprotective agent riluzole, the diuretic ethoxzolamide, the antidiabetic zopolrestat, the immunosuppressant frentizole, or the diagnostic tool thioflavin T (Figure 1A).

The potent antiproliferative effects exerted by benzothiazoles in different cancer models has elicited interest in these molecules as promising antitumor agents (Figure 1B) [5,6]. Considering that 2-phenylbenzothiazoles have been recognized as highly potent cytotoxic compounds, several derivatives were identified and tested in different cancer cell lines. In particular, the chemical manipulation of the lead compound DF203 [7], a 2-phenylbenzothiazole derivative, led to the development of the clinic candidate prodrug Phortress [8]. More recently, the derivative BLZ945, acting as a CSF-1R kinase inhibitor [9,10], has been identified, and it is currently under evaluation as single agent and in combination with spartalizumab for the treatment of advanced solid tumors in adults (ClinicalTrials.gov, NCT02829723) [11]. The 2-ureidobenzothiazole derivative KST016366 has been reported as a potent multikinase inhibitor, displaying a broad-spectrum antiproliferative activity against a wide panel of cancer cell lines [12].

**Figure 2:**
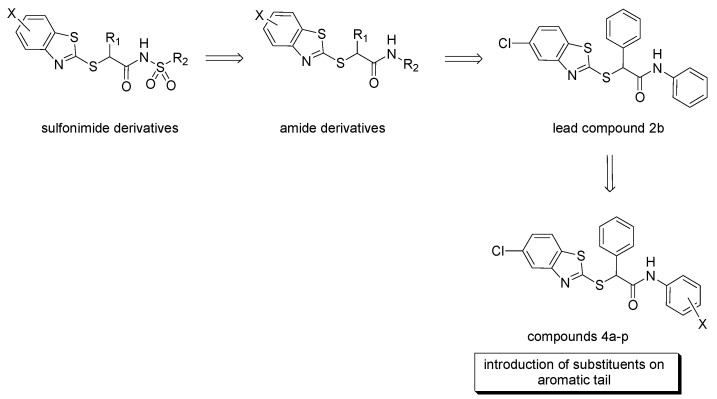
Cytotoxic 2-mercaptobenzothiazole derivatives containing sulfonimide or amide groups, and novel derivatives **4a**–**p** synthesized in this study.

In previous studies, our research group synthesized 2-mercaptobenzothiazole derivatives as Peroxisome Proliferator-Activated Receptor Alpha (PPARα) antagonists [13] (Figure 2). These compounds were able to antagonize PPARα at low micromolar concentrations, and displayed also interesting antiproliferative effects when tested in paraganglioma, glioblastoma, colorectal and pancreatic cancer cell models [14,15]. Interestingly, the benzothiazole derivative **2b** showed a marked cytotoxic effect, mainly in paraganglioma cells, with a dose-dependent inhibition profile and a potency comparable to that of the commercially available PPARα antagonist GW6471 [16,17]. However, it cannot be excluded that other mechanisms of action, or molecular targets, in addition to PPARα inhibition, may emerge for explaining the antiproliferative activity of this compound.

To further explore the potential of this class of molecules as antiproliferative agents, we synthetized novel derivatives of the *lead compound* **2b** by keeping unaltered the benzothiazole scaffold and the amide functional group, and by introducing substituents on the distal aromatic ring. In particular, substituents with different electronic properties and steric hindrance were selected to obtain a series of *para*-substituted (**4a**–**f**), *meta*-substituted (**4g**–**l**) and disubstituted analogs that bear electron-withdrawing substituents (**4m**–**p**). These modifications were performed to test how this molecular portion could modulate the cytotoxic activity in different cancer cell lines.

In this regard, the synthesized compounds **4a**–**p** were tested for their antiproliferative activity in three distinct pancreatic cancer cell lines (AsPC-1, Capan-2, BxPC-3) and two paraganglioma cell lines (PTJ64i, PTJ86i) that have been established by Prof. Cama’s research group at the University of Chieti [17]. Compounds of the series that consistently affected cell viability across the tested cancer cell lines more potently than the *lead compound* **2b** were further analyzed against normal HFF-1 fibroblast cells, to evaluate their toxicity. In the perspective of clinical translation, we also tested whether the most potent and less toxic derivatives could be usefully combined with already approved drugs by analyzing the effects of combined treatments on cancer and normal cell viability.

A target prediction study was also performed to shed light on the putative mechanisms of action contributing to the antiproliferative effects of novel compounds.

## 2. Results and Discussion

### 2.1. Chemistry

**Scheme 1:**
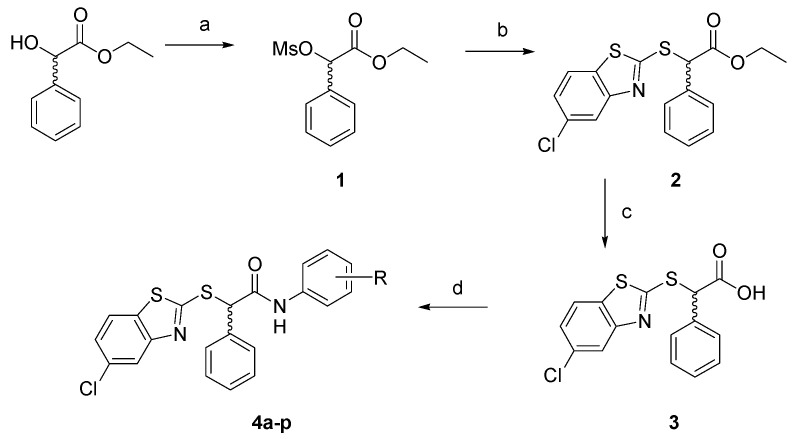
Synthetic route to final compounds **4a**–**p**. Reagents and conditions: (a) mesyl chloride, TEA, THF, 0 °C-r.t.; (b) 5-chloro-2-mercaptobenzothiazole, TEA, THF, 0°-r.t.−50 °C; (c) NaOH 2N, THF, r.t.; (d) substituted aniline, DCC, HOBt, NMM, DMF, 0 °C-r.t.

The synthesis of the planned compounds **4a**–**p** was carried out as depicted in Scheme 1. Briefly, the commercially available ethyl mandelate was treated with mesyl chloride and triethylamine in THF at 0 °C. The resulting mesylate **1** was reacted with 5-chloro-2-mercaptobenzothiazole and triethylamine in THF at 50 °C for 24 h; the ester **2** was hydrolyzed in a basic medium to afford acid **3**. The direct coupling of **3** with the proper amines HOBt, DCC, and *N*-methylmorpholine in DMF led to the amides **4a**–**p**. Crude products were purified by column chromatography or crystallization, obtaining desired amides in good purity and discrete yields.

Final compounds, including para-substituted **4a**–**f**, meta-substituted **4g**–**l** and a group of m–p disubstituted analogues **4m**–**p** bearing electron-withdrawing substituents, are reported in Table 1. For each compound, the method of purification, the yield, and the melting point are also specified.

### 2.2. Antiproliferative Activity

We analyzed by MTT assays the effects of synthesized compounds **4a**–**p** on the viability of three pancreatic cancer cell lines (AsPC-1, Capan-2, BxPC-3) and two paraganglioma cell models (PTJ64i, PTJ86i), based on relevant antiproliferative effects previously shown by the lead compound **2b** in the same cells [16].

**Figure 3:**
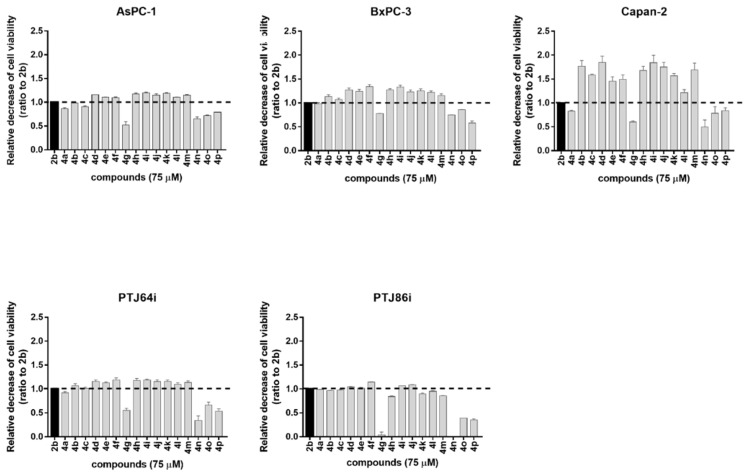
Screening of the effects of novel derivatives (**4a**–**p**) on the viability of pancreatic (AsPC-1, BxPC-3, and Capan-2) and paraganglioma (PTJ64i and PTJ86i) cancer cell lines. The *lead compound* **2b** was included as a reference and the histograms show the relative decrease of cancer cell viability observed after treatments, as compared to **2b**. Cell viability was assessed by an MTT assay using compounds at 75 µM for 72 h. Data shown are the means ± SD of duplicate experiments with quintuplicate determinations and are calculated as ratios relative to the reference compound **2b** (dashed line).

Compounds **4a**–**p** were submitted to a preliminary MTT assay at a one-point screening concentration of 75 μM for 72 h, including **2b** as a reference compound (Figure 3). Overall, the majority of the tested compounds affected cancer cell viability, with a potency comparable, or superior to, **2b** with the exception of **4g** (m-methoxy) and the 2-bromo-substituted derivatives **4n** (2-Br, 5-NO_2_), **4o** (2-Br, 4-CF_3_), and **4p** (2-Br, 5-CF_3_). The presence of the electron-donating methoxy group decreased the activity of **2b**, in both the para and meta positions. Conversely, the introduction of electron-withdrawing substituents, such as halogens, nitro, trifluoromethyl and acetylamino groups, improved the antiproliferative activity of the lead compound **2b**, and this effect was observed for meta and para positions, including the m,p-dichloro derivative **4m**. This general trend was observed for **4a**–**p** in all the selected cancer cell lines. Starting from these data, we selected nine compounds displaying a greater antiproliferative activity than **2b** to perform concentration–response curves. Specifically, these compounds, namely **4d**, **4e**, **4f**, **4h**, **4i**, **4j**, **4k**, **4l**, and **4m**, were tested at concentrations of 3, 6, 12 and 24 µM for 72 h. In addition, the nine compounds, together with **2b**, were tested against human fibroblasts HFF-1, in order to evaluate their selectivity against tumor cells, as compared to normal cells. Effects on cancer or normal cell viability were extrapolated from concentration–response curves and are expressed as IC_50_ values in Table 2.

Notably, the compounds **4k** and **4l** displayed the greatest and most consistent selectivity index (SI) values across the tested cancer cell lines (Table 3 and Table S1), which supported their potential as effective and safe anticancer agents in pancreatic cancer and paraganglioma treatment.

**Figure 4:**
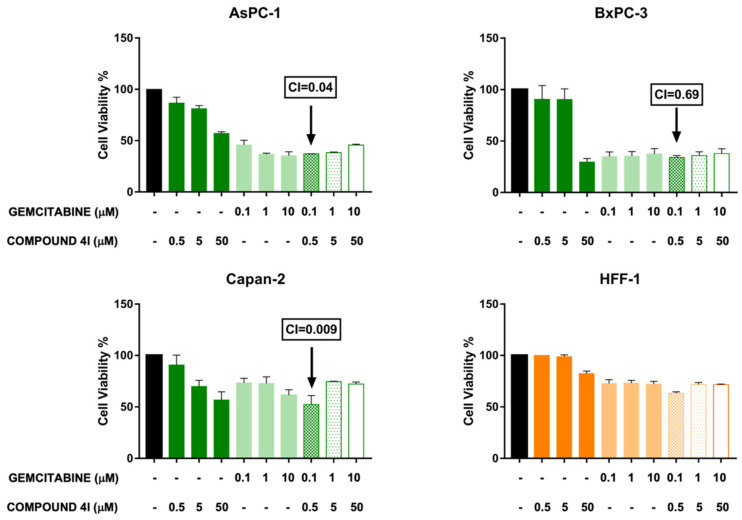
Effect of combined treatments with compound **4l** and gemcitabine on the viability of pancreatic cancer and normal fibroblast cells. Cell viability was assessed by MTT assays after a 72 h incubation of cells with **4l** and gemcitabine at the indicated concentrations, as single agents or in combination. Histograms represent the means ± SD of two independent experiments with quintuplicate determinations. Combination indexes (CIs) were calculated by CompuSyn software. Combinations assessed as synergistic by CIs < 1 are indicated.

Considering that gemcitabine is one of the first-line therapies in pancreatic cancer, to deepen the potential clinical relevance of compounds **4k** and **4l** we further analyzed the effects of the combinations between each of them and gemcitabine on pancreatic cancer cell line viability (Figure 4 and Figure S1, and Table 4). 

Remarkably, the combination between the lowest concentrations of gemcitabine and **4l** (0.1 and 0.5 µM, respectively) decreased pancreatic cancer cell viability in a more marked and synergistic manner (CIs < 1) across the three pancreatic cancer cell lines, as compared to combinations with higher concentrations of the two agents (Figure 4 and Table 4). Notably, the combination with the lowest concentrations of the two compounds appeared safer in normal fibroblast HFF-1 cells, as compared with the pancreatic cancer cell lines (inhibition rate 37% in HFF-1, as compared with 63% in AsPC-1, 66% in BxPC-3 and 48% in Capan-2), supporting the relevance of compound **4l** in the perspective of clinical translation (Table 4). 

### 2.3. Target Prediction Studies

In principle, there could be multiple putative molecular targets for explaining the cytotoxic activity of derivatives **4a**–**p**, and their identification may contribute to a more comprehensive understanding of their bioactivity. Since the high-throughput in vivo target profiling of compounds to identify a potential binding protein for a specific molecule could be expensive and time-consuming, an in silico target fishing study was applied to identify novel proteins possibly involved in the network of molecular events underlying the cytotoxic activity against the cancer cell lines. In silico target fishing (also known as target prediction or target identification) is emerging as an efficient alternative to predict the macromolecular target speedily [18]. A primary distinction divides these methods into ligand-based, i.e., comparing the characteristics (fingerprints) of the query molecule with those present in the databases, and structure-based, i.e., comparing the putative ligand with the features of the target protein’s active site [19]. Various tools, freely accessible or not, have been designed to this aim.

In the present work, all query molecules were analyzed using different web-based tools including ligand-based methods such as SEA SEARCH, PLATO, PPB2, and SuperPred, and structure-based tools such as PharmMapper (Table 5). The resulting targets were filtered, selecting only those expressed in the human organism. Furthermore, as most tools retrieve many possible targets, only the highest-ranked (more reliable) were selected and analyzed. Results for the selected derivative **4l** are shown in Table 6, and the prediction results for other analogue compounds are very similar to those presented.

By analyzing the obtained results, to reduce the risk of false-positive prediction [30] we focused on the target protein classes more frequently retrieved by multiple tools, namely cannabinoid receptors (CBR1, CBR2, and G-protein coupled receptor 55—GPCR55—an orphan GPCR binding cannabinoid), which were predicted by four over six programs, as well as sentrin-specific proteases (SENP6, SENP7, SENP8), which were suggested by three over six programs (Table 6). To further assess the significance of our prediction, the potential role of these two targets in pancreatic cancer was explored by a literature research.

Cannabinoid receptors belong to the Class A GPCR family and regulate several functions, such as neurotransmission, immune and inflammatory responses [31]. It is worth noting that the cannabinoid system is a well-known player in cancer biology [32]. The overexpression of both CB1 and CB2 receptors on pancreatic cancer cells, as compared to a very limited expression in healthy pancreatic cells, was revealed by a study in patients [33]. In addition, antagonists to these receptors seem to be extremely promising as antitumor agents, since they are selective in interacting with pancreatic tumor cells, as compared to healthy cells [34].

Sentrin-specific proteases, also known as SUMO-specific proteases, are cysteine proteases responsible for the DeSUMOylation of target proteins, an essential post-translational modification process [35]. To date, seven SENP isoforms have been identified, showing different localization and substrate preference [36]. Considering that SENPs regulate proteins involved in DNA repair, cell cycle, and neovascularization, the deregulation (overexpression or downregulation) of SENP activity results in cellular dysfunction associated with the development of different diseases, including prostate, thyroid, colon, lung, and pancreatic cancer [37]. For instance, the upregulation of SENP3 is a prognostic marker of pancreatic cancer according to the Cancer Genome Atlas (TCGA) [38]. For these reasons, in recent years SENP proteases have emerged as potential targets for cancer therapy [39]; the high sequence homology of members of human SENPs, along with the differences in substrate specificity and subcellular localization might be an interesting way to discover selective SENP inhibitors [40]. 

### 2.4. Docking Studies

To further validate our predictions, docking calculations were carried out on two representative target proteins. Ligands were flexibly docked in the binding site of the Cannabinoid CB1 receptor (CBR1) using the crystallographic structure of CBR1 retrieved by the Protein Data Bank (PDB: 6KPG) [41].

**Figure 5:**
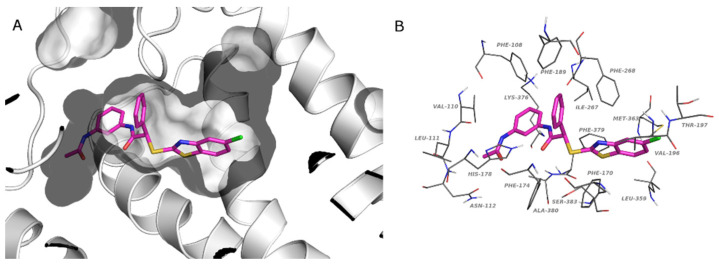
Predicted binding pose of **4l** in the catalytic site of CBR1 (PDB:6KPG). Panel (**A**) CBR1: gray cartoon, binding cavity: gray surface. Panel (**B**) Residues that interact with **4l**: gray lines. (**A**,**B**) Compound **4l,** magenta stick. (O, red; N, blue; S, yellow; Cl, green).

Docked poses in the CBR1 binding site are pretty well conserved among studied compounds: Figure 5a reports the binding mode of compound **4l** highlighting the optimal fitting of the ligand in the CB1 binding pocket. The reported ligand establishes interactions with Phe108, Val110, Leu111, Asn112, Phe170, Phe174, His178, Phe189, Val196, Thr197, Ile267, Phe268, Leu359, Met363, Lys376, Phe379, Ala380, and Ser383 (Figure 5b). These contacts are common to the other ligands as demonstrated by the interaction diagram reported in the Supplementary Materials (Supplementary Figure S2).

**Figure 6:**
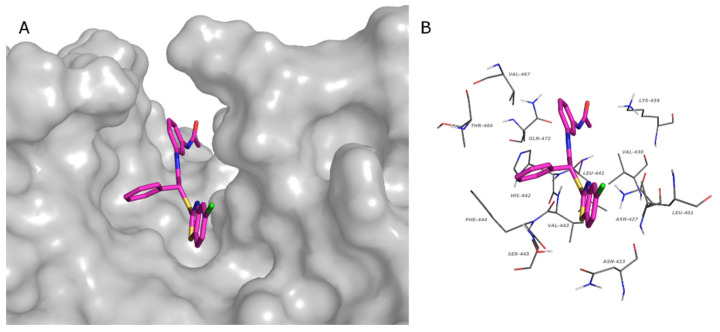
Predicted binding pose of **4l** in the catalytic site of the chimera SENP2-loop1 of SENP6 (PDB:3ZO5). (**A**) Chimera SENP2: gray surface. (**B**) Residues that interact with **4l**: gray lines. (**A**,**B**) Compound **4l,** magenta stick. (O, red; N, blue; S, yellow; Cl, green).

Since the crystallographic structure of SENP6 and SENP7 is not available, the chimeric form of SENP2 containing the SENP6 characteristic loop 1 (similar to SENP7) was used for the structure-based studies (PDB: 3ZO5) [42]. To explore the possible binding sites of the protein, the Sitemap tool was used. Among the identified sites, the one that was superimposable to the most studied SENPs was chosen for the grid generation. This binding site possesses a characteristic form that is able to host all studied compounds. The tridentate structure of the compounds fits well with the structure of the binding site. In Figure 6a, the binding mode of **4l** is shown by the surface representation of the protein, while in Figure 6b, the residues that make contacts with the ligand are reported. In detail, these residues are Leu401, Asn 423, Asn427, Val430, Lys434, Leu441, His442, Val443, Phe444, Ser445, Thr464, Val467, and Gln472. The interaction diagram of all compounds and residues is reported in Supplementary Figure S3. 

Although the identified protein classes seem to be promising targets of our benzothiazole derivatives, further in vitro and in vivo studies will be necessary to validate our prediction.

### 2.5. Physicochemical and Pharmacokinetic Properties Calculation

QikProp [43] calculations were carried out to predict the parameters affecting the drug-likeness and bioavailability of the studied benzothiazoles (Table 7). The calculated physicochemical and pharmacokinetic properties denote that the studied compounds have a drug-like profile, presenting at most one violation of the rule of five due to their lipophilicity (logPoct/water > 5). The predicted low solubility and possible HERG inhibition represent limiting aspects that need further optimization. On the other hand, our compounds present excellent oral absorption and remarkable cell permeability. Moreover, some of the analyzed compounds show good CNS activity and a promising brain/blood partition coefficient.

## 3. Materials and Methods

### 3.1. Chemistry

Melting points were determined with a Buchi Melting Point B-450 and were uncorrected. NMR spectra were recorded on a Varian Mercury 300 spectrometer with ^1^H at 300.060 MHz and ^13^C at 75.475 MHz. Proton chemical shifts were referenced to the TMS internal standard. Chemical shifts are reported in parts per million (ppm, δ units). Coupling constants are reported in units of Hertz (Hz). Splitting patterns are designed as s, singlet; d, doublet; t, triplet; q, quartet; dd, double doublet; m, multiplet; b, broad. Elemental analyses were carried out on a PerkinElmer 240B micro-analyzer, obtaining results within ± 0.4 % of the theoretical values. The purity of all compounds was over 98%. All commercial chemicals and solvents were reagent grade and were obtained from Merck; they were used without further purification, unless otherwise specified. Chemical reactions were monitored by thin layer chromatography on silica gel plates (60F-254, Sigma Aldrich, Italy) and the analysis of the plates was carried out using a UV lamp 254/365 nm. Flash chromatography was performed on silica gel 60 (Merck).

#### 3.1.1. Synthesis of Ethyl 2-[(Methylsulfonyl)oxy]-2-phenylacetate **1**

Triethylamine (834 μL, 6 mmol) was added to a solution of ethyl mandelate (162 mg, 1 mmol) in THF (8 mL) at 0 °C under stirring. Then methanesulfonyl chloride (232 μL, 3 mmol) was added dropwise, and the reaction was allowed to stir for two hours at room temperature. A saturated solution of ammonium chloride was added to the mixture, that was extracted with dichloromethane. Combined organic phases were washed with a saturated sodium chloride solution, dried on sodium sulfate, and the solvent was evaporated under reduced pressure. The crude material was purified by column chromatography on silica gel, with petroleum ether/ethyl acetate 8:2 as eluent. White solid, 93% yield, m.p. 60–61 °C; ^1^H NMR (CDCl_3_) δ 1.23 (t, 3H, *J* 7.2 Hz), 3.08 (s, 3H), 4.16–4.30 (m, 2H), 5.91 (s, 1H), 7.39–7.46 (m, 5H); ^13^C NMR (CDCl_3_) δ 13.9, 39.4, 62.3, 79.0, 127.7, 129.0, 129.9, 132.7, 167.7.

#### 3.1.2. Synthesis of Ethyl 2-[(5-Chlorobenzo[*d*]thiazol-2-yl)thio]-2-phenylacetate **2**

Triethylamine (278 μL, 2 mmol) was added to a solution of 5-cloro-2-mercaptobenzothiazole (202 mg, 1 mmol) in THF (10 mL) at 0 °C under stirring. After 30 min mesylate 1 (258 mg, 1 mmol) was added dropwise, and the temperature was allowed to reach room temperature. The mixture was then heated at 50 °C and stirred for 48 h. After the removal of THF, the resulting oil was dissolved in distilled water and extracted with dichloromethane. The organic phase was washed with a saturated solution of sodium chloride, dried on sodium sulfate, and evaporated under reduced pressure. The crude product was purified by column chromatography on silica gel, with petroleum ether/ethyl acetate 8:1 as eluent. Pale yellow solid, 87% yield; ^1^H NMR (CDCl_3_) δ 1.26 (t, 3H, *J* 6.9 Hz), 4.19 and 4.28 (dq, 2H, *J* 6.9 Hz), 5.76 (s, 1H), 7.25–7.38 (m, 5H), 7.52 (dd, 1H, *J* 8.4, 1.8 Hz), 7.64 (d, 1H, *J* 8.4 Hz), 7.83 (d, 1H, *J* 1.8 Hz); ^13^C NMR (CDCl_3_) δ 14.3, 54.8, 62.5, 121.7, 121.9, 125.0, 128.7, 129.1, 129.2, 132.3, 133.9, 153.9, 167.0, 169.6.

#### 3.1.3. Synthesis of 2-[(5-Chlorobenzo[d]thiazol-2-yl)thio]-2-phenylacetic Acid **3**

NaOH 2N (4 mL, 8 mmol) was added to ester 2 (363 mg, 1 mmol) in THF (5 mL) and the solution was stirred at room temperature overnight. THF was removed under reduced pressure, and the aqueous phase was acidified by HCl 2N, obtaining a precipitate that was collected by filtration under vacuum and recrystallized from cyclohexane. White crystals, 99% yield, m.p. 186–188 °C; ^1^H NMR (CD_3_OD) δ 5.79 (s, 1H), 7.31–7.41 (m, 5H), 7.54 (dd, 1H, *J* 8.4, 1.8 Hz), 7.81 (d, 1H, *J* 8.4 Hz), 7.84 (d, 1H, *J* 1.8 Hz); ^13^C NMR (CD_3_OD) δ 55.0, 120.9, 122.2, 124.8, 128.4, 128.7, 128.9, 132.2, 133.8, 135.0, 153.8, 167.9, 171.2.

#### 3.1.4. General Procedure for the Synthesis of Amides **4a**–**p**

To a stirred solution of acid 3 (168 mg, 0.5 mmol) in DMF (5 mL) at 0 °C, *N*,*N*’-dicyclohexylcarbodiimide (DCC, 103 mg, 0.5 mmol) and hydroxybenzotriazole (HOBt, 77 mg, 0.5 mmol) were added. After 15 min, *N*-methylmorpholine (NMM, 55 μL, 0.5 mmol) and the selected amine (0.5 mmol) were added in sequence to the reaction mixture. The resulting solution was then stirred at room temperature for 24 h, concentrated and the residue was dissolved in dichloromethane, and washed with a saturated solution of sodium bicarbonate and brine. The combined organic layers were dried on sodium sulfate and concentrated under *vacuum* to provide the crude products, which were purified by column chromatography or crystallization.

##### 2-[(5-Chlorobenzo[*d*]thiazol-2-yl)thio]-*N*-(4-methoxyphenyl)-2-phenylacetamide **4a**

Pale yellow solid (silica gel, chloroform), 58% yield; m.p. 190 °C (dec); ^1^H NMR (CDCl_3_) δ 3.76 (s, 3H), 5.81 (s, 1H), 6.82 (d, 2H, *J* 8.7 Hz), 7.30–7.42 (m, 6H), 7.54–7.57 (m, 2H), 7.67 (d, 1H, *J* 8.4 Hz), 7.91 (d, 1H, *J* 1.8 Hz), 9.01 (bs, 1H); ^13^C NMR (CDCl_3_) δ 55.0, 55.4, 114.1, 121.1, 121.4, 121.9, 125.3, 128.8, 128.9, 129.0, 130.8, 132.6, 133.5, 134.5, 152.9, 159.5, 166.4, 168.3. Calcd for C_22_H_17_ClN_2_O_2_S_2_: C, 59.92; H, 3.89; N, 6.35. Found: C, 59.81; H, 3.90; N, 6.33.

##### 2-[(5-Chlorobenzo[*d*]thiazol-2-yl)thio]-*N*-(4-chlorophenyl)-2-phenylacetamide **4b**

Pale yellow solid (silica gel, chloroform), 63% yield; m.p. 178–180 °C; ^1^H NMR (CDCl_3_) δ 5.83 (s, 1H), 7.26 (d, 1H, *J* 8.7 Hz), 7.34–7.56 (m, 9H), 7.70 (d, 1H, *J* 8.7 Hz), 7.95 (d, 1H, *J* 1.8 Hz), 9.39 (bs, 1H); ^13^C NMR (CDCl_3_) δ 54.8, 120.9, 121.0, 125.5, 128.8, 129.0, 129.1, 129.5, 132.7, 133.5, 134.1, 136.3, 152.8, 166.9, 168.5. Calcd for C_21_H_14_Cl_2_N_2_OS_2_: C, 56.63; H, 3.17; N, 6.29. Found: C, 56.77; H, 3.18; N, 6.27.

##### 2-[(5-Chlorobenzo[*d*]thiazol-2-yl)thio]-*N*-(4-fluorophenyl)-2-phenylacetamide **4c**

Pale yellow solid (silica gel, dichloromethane), 52% yield; m.p. 168–170 °C; ^1^H NMR (CDCl_3_) δ 5.77 (s, 1H), 6.98 (t, 2H, *J* 8.4 Hz), 7.32–7.55 (m, 8H), 7.67 (d, 1H, *J* 8.7 Hz), 7.91 (d, 1H, *J* 2.4 Hz), 9.23 (bs, 1H); ^13^C NMR (CDCl_3_) δ 54.8, 115.5, 115.8, 121.1, 121.3, 121.5, 122.0, 125.4, 128.8, 128.9, 129.0, 132.6, 133.7, 134.2, 153.1, 157.9, 161.4, 166.8. Calcd for C_21_H_14_ClFN_2_OS_2_: C, 58.80; H, 3.29; N, 6.53. Found: C, 58.69; H, 3.30; N, 6.54.

##### 2-[(5-Chlorobenzo[*d*]thiazol-2-yl)thio]-2-phenyl-*N*-[4-(trifluoromethyl)phenyl] Acetamide **4d**

Pale yellow solid (silica gel, dichloromethane), 47% yield; m.p. 183–185 °C; ^1^H NMR (CDCl_3_) δ 5.79 (s, 1H), 7.34–7.41 (m, 4H), 7.51–7.56 (m, 4H), 7.64 (d, 2H, *J* 8.1 Hz), 7.70 (d, 1H, *J* 8.1 Hz), 7.93 (d, 1H, *J* 1.5 Hz), 9.71 (bs, 1H); ^13^C NMR (CDCl_3_) δ 54.6, 119.2, 121.0, 122.1, 125.6, 126.3 (q), 128.9, 129.0, 132.8, 133.5, 133.8, 140.8, 152.7, 167.3, 168.7. Calcd for C_22_H_14_ClF_3_N_2_OS_2_: C, 55.17; H, 2.95; N, 5.85. Found: C, 55.22; H, 2.94; N, 5.84.

##### 2-[(5-Chlorobenzo[*d*]thiazol-2-yl)thio]-*N*-(4-nitrophenyl)-2-phenylacetamide **4e**

Pale yellow solid (silica gel, dichloromethane), 43% yield, m.p. 191–193 °C; ^1^H NMR (CDCl_3_) δ 5.79 (s, 1H), 6.61 (d, 2H, *J* 9.3 Hz), 7.33–7.41 (m, 4H), 7.50–7.54 (m, 2H), 7.70 (d, 2H, *J* 8.7 Hz), 7.91 (d, 1H, *J* 1.8 Hz), 8.05 (d, 2H, *J* 9 Hz), 8.18 (d, 2H, *J* 9.3 Hz), 10.05 (bs, 1H); ^13^C NMR (CDCl_3_) δ 54.5, 113.3, 119.1, 120.9, 122.2, 125.1, 125.7, 126.3, 128.9, 129.1, 129.2, 133.0, 133.4, 143.6, 143.7, 152.5, 167.6, 168.9. Calcd for C_21_H_14_ClN_3_O_3_S_2_: C, 55.32; H, 3.09; N, 9.22. Found: C, 55.57; H, 3.10; N, 9.24.

##### *N*-(4-Acetamidophenyl)-2-[(5-chlorobenzo[*d*]thiazol-2-yl)thio]-2-phenylacetamide **4f**

White cristals (from ethyl acetate/methanol), 59% yield; m.p. 243 °C (dec); ^1^H NMR (DMSO-*d6*) δ 1.98 (s, 3H), 5.96 (s, 1H), 7.29–7.51 (m, 8H), 7.64–7.69 (m, 2H,), 7.86 (d, 1H, *J* 2.4 Hz), 8.03 (d, 1H, *J* 8.4 Hz), 9.89 (bs, 1H) 10.64 (bs, 1H); ^13^C NMR (DMSO-*d6*) δ 24.3, 42.5, 110.0, 119.7, 120.2, 121.0, 123.9, 125.2, 128.7, 129.2, 131.7, 134.0, 135.9, 136.5, 153.7, 166.4, 167.0, 168.0, 168.4. Calcd for C_23_H_18_ClN_3_O_2_S_2_: C, 59.03; H, 3.88; N, 8.98. Found: C, 59.08; H, 3.87; N, 8.96.

##### 2-[(5-Chlorobenzo[*d*]thiazol-2-yl)thio]-*N*-(3-methoxyphenyl)-2-phenylacetamide **4g**

White solid (silica gel, cyclohexane/ethyl acetate 7:1), 44% yield; m.p. 160–162 °C; ^1^H NMR (CDCl_3_) δ 3.75 (s, 3H), 5.78 (s, 1H), 6.64 (dd, 1H, *J* 8.1, 2.4 Hz), 6.92 (d, 1H, *J* 8.1 Hz), 7.17 (t, 1H, *J* 8.1 Hz), 7.30–7.43 (m, 5H), 7.53 (dd, 2H, *J* 8.1, 2.4 Hz), 7.67 (d, 1H, *J* 8.1 Hz), 7.93 (d, 1H, *J* 1.8 Hz), 9.30 (bs, 1H); ^13^C NMR (CDCl_3_) δ 54.8, 55.2, 104.9, 110.8, 111.6, 121.1, 122.0, 125.3, 128.8, 128.9, 129.0, 129.7, 132.6, 133.6, 134.2, 139.0, 153.0, 160.1, 166.8, 168.4. Calcd for C_22_H_17_ClN_2_O_2_S_2_: C, 59.92; H, 3.89; N, 6.35. Found: C, 59.80; H, 3.89; N, 6.37.

##### 2-[(5-Chlorobenzo[*d*]thiazol-2-yl)thio]-*N*-(3-chlorophenyl)-2-phenylacetamide **4h**

White solid (silica gel, dichloromethane), 76% yield; m.p. 175–177 °C; ^1^H NMR (CDCl_3_) δ 5.77 (s, 1H), 7.08 (d, 1H, *J* 8.1 Hz), 7.20–7.41 (m, 6H), 7.52 (dd, 2H, *J* 8.1, 2.4 Hz), 7.69 (d, 1H, *J* 8.1 Hz), 7.72 (s, 1H), 7.93 (d, 1H, *J* 1.8 Hz), 9.42 (bs, 1H); ^13^C NMR (CDCl_3_) δ 54.7, 171.5, 119.8, 121.1, 122.0, 124.5, 125.5, 128.8, 129.0, 129.1, 130.0, 132.8, 133.5, 133.9, 134.7, 138.9, 152.8, 167.0. Calcd for C_21_H_14_Cl_2_N_2_OS_2_: C, 56.63; H, 3.17; N, 6.29. Found: C, 56.67; H, 3.16; N, 6.27.

##### 2-[(5-Chlorobenzo[*d*]thiazol-2-yl)thio]-*N*-(3-fluorophenyl)-2-phenylacetamide **4i**

White solid (silica gel, dichloromethane), 51% yield; m.p. 151–153 °C; ^1^H NMR (CDCl_3_) δ 5.77 (s, 1H), 6.79 (dt, 1H), 7.08–7.55 (m, 9H), 7.68 (d, 1H, *J* 8.1 Hz), 7.92 (d, 1H, *J* 2.4 Hz), 9.44 (bs, 1H); ^13^C NMR (CDCl_3_) δ 54.8, 107.1, 107.4, 111.1, 111.4, 114.8, 114.9, 121.1, 122.0, 125.4, 128.8, 128.9, 129.0, 130.0, 130.1, 132.7, 133.6, 134.1, 139.2, 152.9, 161.3, 164.5, 167.1. Calcd for C_21_H_14_ClFN_2_OS_2_: C, 58.80; H, 3.29; N, 6.53. Found: C, 58.63; H, 3.30; N, 6.55.

##### 2-[(5-Chlorobenzo[*d*]thiazol-2-yl)thio]-2-phenyl-*N*-[3-(trifluoromethyl) phenyl]acetamide **4j**

White solid (silica gel, cyclohexane/diethyl ether 4:1), 45% yield; m.p. 155–157 °C; ^1^H NMR (CDCl_3_) δ 5.77 (s, 1H), 7.33–7.41 (m, 7H), 7.53 (d, 2H, *J* 6.3 Hz), 7.61 (d, 1H, *J* 8.1 Hz), 7.70 (d, 1H, *J* 8.7 Hz), 7.93 (s, 2H); ^13^C NMR (CDCl_3_) δ 54.6, 116.4 (q), 112.0, 121.0, 122.0, 122.4, 125.5, 128.8, 129.0, 129.5, 132.8, 133.6, 133.8, 138.3, 152.9, 167.2. Calcd for C_22_H_14_ClF_3_N_2_OS_2_: C, 55.17; H, 2.95; N, 5.85. Found: C, 55.21; H, 2.96; N, 5.83.

##### 2-[(5-Chlorobenzo[*d*]thiazol-2-yl)thio]-*N*-(3-nitrophenyl)-2-phenylacetamide **4k**

Pale yellow solid (silica gel, dichloromethane), 48% yield; m.p. 193–195 °C; ^1^H NMR (CDCl_3_) δ 5.77 (s, 1H), 7.34–7.54 (m, 7H), 7.70 (d, 1H, *J* 8.7 Hz), 7.83 (dd, 1H, *J* 8.1, 1.2 Hz), 7.92–7.95 (m, 2H), 8.47 (t, 1H, *J* 2.1 Hz), 9.88 (bs, 1H); ^13^C NMR (CDCl_3_) δ 54.6, 110.0, 114.5, 119.0, 121.0, 122.1, 125.1, 125.7, 128.8, 129.1, 129.8, 133.0, 133.6, 139.0, 148.5, 167.4. Calcd for C_21_H_14_ClN_3_O_3_S_2_: C, 55.32; H, 3.09; N, 9.22. Found: C, 55.45; H, 3.08; N, 9.22.

##### *N*-(3-Acetamidophenyl)-2-[(5-chlorobenzo[*d*]thiazol-2-yl)thio]-2-phenylacetamide **4l**

White crystals (from chloroform), 41% yield; m.p. 197–199 °C; ^1^H NMR (CDCl_3_) δ 2.11 (s, 3H), 5.76 (s, 1H), 7.21 (d, 2H, *J* 5.4 Hz), 7.25–7.39 (m, 6H), 7.52 (dd, 2H, *J* 8.1, 2.4 Hz), 7.65 (d, 1H, *J* 8.7 Hz), 7.80 (s, 1H), 7.94 (d, 1H, *J* 1.8 Hz), 9.18 (bs, 1H); ^13^C NMR (CDCl_3_) δ 24.6, 55.2, 110.0, 110.9, 115.2, 115.7, 121.4, 121.9, 125.3, 128.8, 128.9, 129.0, 129.6, 132.6, 133.6, 134.2, 138.2, 138.5, 153.1, 166.9, 168.4. Calcd for C_23_H_18_ClN_3_O_2_S_2_: C, 59.03; H, 3.88; N, 8.98. Found: C, 59.08; H, 3.89; N, 9.00.

##### 2-[(5-Chlorobenzo[d]thiazol-2-yl)thio]-N-(3,4-dichlorophenyl)-2-phenyl Acetamide **4m**

White solid (silica gel, dichloromethane), 44% yield; m.p. 203–204 °C; ^1^H NMR (DMSO-*d_6_*) δ 5.96 (s, 1H), 7.33–7.47 (m, 5H), 7.54–7.64 (m, 3H), 7.85 (d, 1H, *J* 1.5 Hz), 7.94 (d, 1H, *J* 2.4 Hz), 8.03 (d, 1H, *J* 8.1 Hz), 11.03 (bs, 1H); ^13^C NMR (DMSO-*d_6_*) δ 56.2, 119.8, 120.9, 123.9, 125.1, 125.9, 128.7, 129.2, 129.4, 131.3, 131.6, 131.7, 134.0, 135.7, 138.9, 153.6, 167.3, 167.7. Calcd for C_21_H_13_Cl_3_N_2_OS_2_: C, 52.57; H, 2.73; N, 5.84. Found: C, 52,44; H, 2.72; N, 5.85.

##### *N*-(2-Bromo-5-nitrophenyl)-2-[(5-chlorobenzo[*d*]thiazol-2-yl)thio]-2-phenyl Acetamide **4n**

White crystals (from cyclohexane/methanol), 48% yield; m.p. 176–178 °C; ^1^H NMR (CDCl_3_) δ 5.98 (s, 1H), 7.31–7.57 (m, 7H), 7.69 (d, 1H, *J* 8.7 Hz), 7.87–7.92 (m, 2H), 9.32 (d, 1H, *J* 3 Hz), 9.51 (bs, 1H); ^13^C NMR (CDCl_3_) δ 54.5, 116.7, 119.4, 121.5, 122.0, 125.4, 128.8, 129.2, 129.3, 129.6, 132.6, 133.5, 133.6, 135.5, 152.9, 167.7 Calcd for C_21_H_13_BrClN_3_O_3_S_2_: C, 47.16; H, 2.45; N, 7.86. Found: C, 47.07; H, 2.46; N, 7.85.

##### *N*-[2-Bromo-4-(trifluoromethyl)phenyl]-2-[(5-chlorobenzo[*d*]thiazol-2-yl)thio]-2-phenylacetamide **4o**

White crystals (from petroleum ether/methanol), 51% yield; m.p. 179–180 °C; ^1^H NMR (CDCl_3_) δ 5.95 (s, 1H), 7.24–7.28 (m, 1H), 7.34–7.44 (m, 3H), 7.53–7.60 (m, 3H), 7.67 (d, 1H, *J* 8.7 Hz), 7.75 (s, 1H), 7.91 (d, 1H, *J* 1.8 Hz), 8.49 (d, 1H, *J* 8.7 Hz), 9.14 (bs, 1H); ^13^C NMR (CDCl_3_) δ 55.5, 113.3, 121.6, 121.7, 121.8, 125.3, 125.5, 125.6, 128.7, 129.2, 129.3, 129.4, 132.4, 133.6, 133.8, 153.2, 166.6, 167.3. Calcd for C_22_H_13_BrClF_3_N_2_OS_2_: C, 47.37; H, 2.35; N, 5.02. Found: C, 47.39; H, 2.35; N, 5.01.

##### *N*-[2-Bromo-5-(trifluoromethyl)phenyl]-2-[(5-chlorobenzo[*d*]thiazol-2-yl)thio]-2-phenylacetamide **4p**

White crystals (from petroleum ether), 46% yield; m.p. 157–159 °C; ^1^H NMR (CDCl_3_) δ 5.96 (s, 1H), 7.20–7.32 (m, 2H), 7.39–7.44 (m, 3H), 7.56 (dd, 2H, *J* 7.5, 1.2 Hz), 7.61 (d, 1H, *J* 8.1 Hz), 7.67 (d, 1H, *J* 8.7 Hz), 7.92 (d, 1H, *J* 1.8 Hz), 8.67 (s, 1H), 9.16 (bs, 1H); ^13^C NMR (CDCl_3_) δ 55.2, 119.0, 121.7, 121.8, 121.9, 122.0, 125.2, 128.7, 129.2, 129.3, 132.4, 132.8, 133.9, 136.1, 153.2, 167.4. Calcd for C_22_H_13_BrClF_3_N_2_OS_2_: C, 47.37; H, 2.35; N, 5.02. Found: C, 47.27; H, 2.35; N, 5.04.

### 3.2. Cell Lines, Treatments, and Cell Viability Assay

Pancreatic cancer (AsPC-1, BxPC-3, and Capan-2), paraganglioma (PTJ86i and PTJ64i) and normal fibroblast (HFF-1) cell lines were cultured as previously described [17]. All compounds were dissolved in DMSO (stock solutions) and then diluted in culture media to the final working concentrations. In this way, the solutions were completely clear and devoid of any undissolved material by microscopic inspection. The final concentration of DMSO in the experiments was at most 0.18% and showed no cell toxicity. The effects of compounds 4a-p and 2b on cancer cell viability were tested by an MTT assay (Sigma-Aldrich, St. Louis, MO, USA), as described by Florio et al. [17]. In the initial screening all compounds were tested at a one-point screening concentration of 75 μM for 72 h (five replica wells per each condition). Then, concentration–response curves were generated by incubating cancer or normal fibroblast cell lines for 72 h with compounds showing a greater antiproliferative activity than 2b, at concentrations ranging from 0 μ M to 24 μM (five replica wells per each condition). For combined treatments, the experimental design was made according to the Chou–Talalay method for drug combination studies, as previously described [44].

### 3.3. Calculation of Half Maximal Inhibitory Concentration (IC_50_), Selectivity Index (SI), Combination Index (CI) Values and Statistical Analysis

IC_50_ values were extrapolated from concentration–response curves and calculated using the CompuSyn software [45]. The interactions between compound 4l and gemcitabine were assessed by calculating the CI values using the CompuSyn software. Based on this analysis, a CI < 1 indicates synergism, a CI = 1 indicates additive effects and a CI > 1 indicates antagonism. SI values were calculated as previously described [46]. Comparisons of mean values were performed using an unpaired Student’s *t*-test. For multiple comparisons, a one-way ANOVA followed by Dunnett’s test were employed. A *p*-value < 0.05 was estimated as statistically significant.

### 3.4. In Silico Studies

The SMILE string of the selected compound was obtained from Marvin Sketch (ChemAxon). Swiss Target Prediction database (http://www.swisstargetprediction.ch/), PLATO (http://platomussel.uniba.it/), SEASearch (https://sea.bkslab.org/), PPB2 (http://ppb2.gdb.tools/), SuperPred (https://prediction.charite.de/subpages/target_prediction.php), ChemMapper (http://www.lilab-ecust.cn/chemmapper), and PharmMapper (http://www.lilab-ecust.cn/pharmmapper/) were used to predict potential targets. “Homo sapiens” as the organism and “cancer target” were chosen as an option when possible (SuperPred). In all other cases, the predicted targets were screened by UniProt database (http://www.uniprot.org/uniprot/) by entering the target protein names and selecting only those belonging the “Homo sapiens (Human)” organism and “cancer” as protein name or tissue. Finally, the targets that did not meet the setting parameters were removed. Since these programs use different methods to score the targets, only a max of 20 targets was taken into consideration. All used programs were accessed on 1 March 2022.

The structure-based analysis was carried out using the Schrödinger Life-Sciences Suite 2021–4 [43]. The 2D sketcher in Maestro was used to construct ligand structures that were submitted to LigPrep to obtain ligand 3D geometry, identify all potential tautomers, and protonation states at pH 7.0 ± 0.4. The resulting structures were minimized utilizing MacroModel, the OPLS4 force field, and 5000 steps of the PRCG algorithm with a convergence criterion of 0.05 KJ/mol. Docking calculations were carried out on the 3D coordinates of CBR1 with PDB ID 6KPG and the chimeric form of SENP2 containing the SENP7 loop, PDB ID 3ZO5. Before docking calculations, protein structures were prepared using the Protein Preparation routine in Maestro [47] that fixes the protein structure and relaxes it through a constrained minimization. For the CBR1 the docking protocol was validated by reobtaining the X-ray geometry of the crystallographic ligand (RMSD 0.5135Å). To define the more proper binding site in SENP6, a SiteMap [48,49] calculation was carried out and the subsequent Glide Grid was generated on the predicted site. Glide [50,51] SP flexible docking calculations were carried out on both proteins

## 4. Conclusions

In conclusion, a library of phenylacetamide derivatives containing the benzothiazole nucleus was synthesized and tested for its antiproliferative activity in three pancreatic and two paraganglioma cancer cell lines. Most of the derivatives showed an improved antiproliferative activity, as compared to the *lead compound* 2b, allowing us to trace some preliminary structure–activity relationships. Considering both the antiproliferative activities and selectivity profiles, **4l** was further analyzed in combination with gemcitabine on the three pancreatic cancer cell lines. Notably, the combination of the two compounds when used at the lowest concentrations (0.1 μM gemcitabine and 0.5 μM **4l**, respectively) affected pancreatic cancer cell viability in a potent and synergistic manner. This synergistic effect supports the interest in this class of benzothiazoles in view of a possible translation into cancer treatment. A computational study was also conducted to predict the putative targets involved in the observed antiproliferative activity in pancreatic cancer cells: the different tools used to this aim identified the cannabinoid receptors and the sentrin-specific proteases as potential targets contributing to the bioactivity of this family of compounds. Docking studies on representative targets (CBR1 and SENP6) show that all compounds fit well in their binding site. Future in vitro and in vivo studies will be necessary to validate the predicted molecular targets of our compounds and gain novel insights into the underexplored potential of these molecules as antitumor agents in paraganglioma and pancreatic cancer treatment.

## Figures and Tables

**Table 1:** Final compounds **4a–p** synthesized in this study.

Cpd	R	Purification Conditions	Yield %	m.p.
**4a**	*p*-OCH_3_	Silica gel, eluent chloroform	58	190 °C (dec)
**4b**	*p*-Cl	Silica gel, eluent chloroform	63	178–180 °C
**4c**	*p*-F	Silica gel, eluent dichloromethane	52	168–170 °C
**4d**	*p*-CF_3_	Silica gel, eluent dichloromethane	47	183–185 °C
**4e**	*p*-NO_2_	Silica gel, eluent dichloromethane	43	191–193 °C
**4f**	*p*-NHCOCH_3_	Crystallization from ethyl acetate/methanol	59	243 °C (dec)
**4g**	*m*-OCH_3_	Silica gel, eluent cyclohexane/ethyl acetate 7:1	44	160–162 °C
**4h**	*m*-Cl	Silica gel, eluent dichloromethane	76	175–177 °C
**4i**	*m*-F	Silica gel, eluent dichloromethane	51	151–153 °C
**4j**	*m*-CF_3_	Silica gel, eluent cyclohexane/diethyl ether 4:1	45	155–157 °C
**4k**	*m*-NO_2_	Silica gel, eluent dichloromethane	48	193–195 °C
**4l**	*m*-NHCOCH_3_	Crystallization from chloroform	41	197–199 °C
**4m**	3,4-diCl	Silica gel, eluent dichloromethane	44	203–204 °C
**4n**	2-Br, 5-NO_2_	Crystallization from cyclohexane/methanol	48	176–178 °C
**4o**	2-Br, 4-CF_3_	Crystallization from petroleum ether/methanol	51	179–180 °C
**4p**	2-Br, 5-CF_3_	Crystallization from petroleum ether	46	157–159 °C

**Table 2:** IC_50_ values of **2b** and its nine most active derivatives on cancer and normal cell lines.

	IC_50_ (µM)					
	Pancreatic Cancer			Paraganglioma		Normal Cells
	AsPC-1	BxPC-3	Capan-2	PTJ64i	PTJ86i	HFF-1
**2b**	12.44	14.99	19.65	8.49	16.70	21.37
**4d**	7.66	3.99	8.97	6.79	12.39	9.23
**4e**	12.77	10.69	14.11	9.81	18.87	16.69
**4f**	10.04	18.85	20.10	12.34	12.82	6.54
**4h**	12.16	11.99	17.67	7.27	16.58	11.55
**4i**	14.80	18.60	28.50	8.60	11.70	15.00
**4j**	9.53	13.96	24.18	11.20	17.46	18.10
**4k**	10.08	11.92	16.87	7.47	13.51	23.33
**4l**	14.78	13.67	33.76	10.13	19.88	67.07
**4m**	8.49	9.81	13.33	7.84	19.92	10.32

Note: IC_50_ values for **2b** in paraganglioma cells were previously published [17].

**Table 3:** Selectivity index values for compounds **4k** and **4l**.

	Selectivity Index (SI) Values				
	Pancreatic Cancer			Paraganglioma	
	AsPC-1	BxPC-3	Capan-2	PTJ64i	PTJ86i
**4k**	2.31	1.96	1.38	3.12	1.73
**4l**	4.54	4.91	1.99	6.62	3.37

**Table 4:** Percentage of cell viability inhibition after compound **4l** and gemcitabine treatments in PC and normal fibroblast cell lines.

	Inhibition Rate of Cell Viability, %			
	Pancreatic Cancer			Normal Cells
	AsPC-1	BxPC-3	Capan-2	HFF-1
**4l–0.5 µM**	13.40	6.00	9.34	0.00
**4l–5 µM**	18.87	9.54	30.25	1.00
**4l–50 µM**	42.97	70.50	43.30	17.69
**GEM—0.1 µM**	54.21	65.02	26.87	27.39
**GEM—1 µM**	63.25	64.65	27.22	25.43
**GEM—10 µM**	64.63	62.50	38.65	26.27
**4l (0.5 µM) + GEM (0.1 µM)**	62.85	65.98	47.66	36.69
**4l (5 µM) + GEM (1 µM)**	61.70	64.01	25.40	28.16
**4l (50 µM) + GEM (10 µM)**	54.27	64.09	27.79	25.19

**Table 5:** Description, database, and target ranking criterion of the used web tools. The URL of each tool is indicated. All sites were accessed in 1 March 2022.

Web Tool	Description	Database	Target ranking	URL
**SwissTargetPrediction** [20]	A combination of 2D and 3D similarities with known ligands	ChEMBL23	Similarity threshold of compounds	http://www.swisstargetprediction.ch
**PLATO** [21,22,23,24]	Multifingerprint Similarity Predictive Approach	ChEMBL30	Similarity between query molecule and known target ligands using different fingerprints	http://plato.uniba.it/
**SEA Search** [25]	Similarity searching	ChEMBL27	E-value indicating the reliability of the prediction	https://sea.bkslab.org/
**PPB2** [26]	Similarity searching combined with Machine Learning models	ChEMBL22	Score calculated by the applied model	http://ppb2.gdb.tools/
**SuperPred** [27]	Similarity searching by ECFP4 fingerprints	ChEMBL29	Similarity between query molecule and known target ligands	https://prediction.charite.de/subpages/target_prediction.php
**ChemMapper** [28]	Pharmacophore/shape superposition and statistical background distribution	database of 300M drug-like compounds (ChEMBL, BindingDB, DrugBank, KEGG, PDB)	Similarity between query molecule and known target ligands	http://www.lilab-ecust.cn›chemmapper
**PharmMapper** [29]	Reverse Pharmacophore screening	TargetBank DrugBank, BindingDB and PDTD.	Z-score based on fit score (match feature types and positions)	http://www.lilab-ecust.cn/pharmmapper/

**Table 6:** Predicted targets for compound **4l**.

PLATO	SwissTargetPrediction	SEA	PPB2	SuperPRED	PharmMAPPER	ChemMapper
Peroxisome proliferator-activated receptor alpha	Dual specificity mitogen-activated protein kinase1	Potassium voltage-gated channel subfamily B member 2	Arachidonate 5-lipoxygenase	Glutaminase kidney isoform, mitochondrial	Cbp/p300-intE4:E27	Voltage-dependent T-type calcium channel subunit alpha-1H
Cathepsin K	ATP-binding cassette sub-family G member 2	Neuronal calcium sensor 1	Peroxisome proliferator-activated receptor alpha	Casein kinase II alpha/beta	Coagulation factor XIII A chain	G-protein coupled receptor 55
Cathepsin L	Voltage-gated potassium channel subunit Kv1.5	Ubiquitin carboxyl-terminal hydrolase BAP1	G-protein coupled receptor 55	Muscarinic acetylcholine receptor M3	Cold shock domain-containing protein E1	Cannabinoid receptor 2
Tyrosine-protein kinase LCK	Insulin receptor	Survival motor neuron protein	Cannabinoid CB1 receptor	ADAM10	Short-chain specific acyl-CoA dehydrogenase, mitochondrial	Cannabinoid receptor 1
C-C chemokine receptor type 3	Cannabinoid receptor 1	Potassium channel subfamily K member 9	Cannabinoid CB2 receptor	Aurora kinase B/Inner centromere protein	Homeobox protein Hox-B13	DNA dC->dU-editing enzyme APOBEC-3G
G-protein coupled receptor 55	ALK tyrosine kinase receptor	Cysteinyl leukotriene receptor 1	Vascular endothelial growth factor receptor 2	Caspase-8	Disheveled-associated activator of morphogenesis 1	Probable DNA dC->dU-editing enzyme APOBEC-3A
Carboxy-terminal domain RNA polymerase II polypeptide A small phosphatase 1	Receptor protein-tyrosine kinase erbB-4	Glutamate receptor ionotropic, kainate 1	Coagulation factor X	DNA topoisomerase I	Protection of telomeres protein 1	E3 ubiquitin-protein ligase Mdm2
11-beta-hydroxysteroid dehydrogenase 1	Peroxisome proliferator-activated receptor alpha	6-phosphofructo-2-kinase/fructose-2,6-bisphosphatase 4	Sentrin-specific protease 7	Galectin-3	Regulator of G-protein signaling 6	Eukaryotic translation initiation factor 4H
G-protein coupled receptor 35	MAP kinase p38 alpha	Sentrin-specific protease 8	Epidermal growth factor receptor erbB1	Indoleamine 2,3-dioxygenase	Heterogeneous nuclear ribonucleoprotein R	Polyadenylate-binding protein 1
Sentrin-specific protease 7	c-Jun *N*-terminal kinase 2	Sentrin-specific protease 7	Tyrosine-protein kinase SRC	Sphingosine kinase 1	Calpain-9	MCOLN3 protein
PI3-kinase p110-alpha subunit	Cyclin-dependent kinase 4	Probable G-protein coupled receptor 139	Beta-secretase 1	Adenosine A3 receptor	Glycogen phosphorylase, liver form	Estrogen receptor
Caspase-3	Serine/threonine-protein kinase AKT	Free fatty acid receptor 2	Adenosine A3 receptor	Integrin alpha-V/beta-3	Transcription initiation factor TFIID subunit 13	Putative hexokinase HKDC1
Cannabinoid CB2 receptor	Vascular endothelial growth factor receptor 2	Sentrin-specific protease 6	Dopamine D2 receptor	DNA (cytosine-5)-methyltransferase 1	Proto-oncogene tyrosine-protein kinase Fes/Fps	Hexokinase-1
C-C chemokine receptor type 1	Ribosomal protein S6 kinase alpha 3	Solute carrier family 22 member 6	Serine/threonine-protein kinase Aurora-A	Muscarinic acetylcholine receptor M5	Ig gamma-1 chain C region secreted form	Coagulation factor XII
Sentrin-specific protease 6	Phosphodiesterase 10A	Acyl-CoA (8–3)-desaturase	Vanilloid receptor	Protein-tyrosine phosphatase 2C	Cytochrome P450 2E1	Glyceraldehyde-3-phosphate dehydrogenase
Sentrin-specific protease 8	Pregnane X receptor	Trypsin-3	Induced myeloid leukemia cell differentiation protein Mcl-1	Muscarinic acetylcholine receptor M1	Threonine dehydratase biosynthetic	Induced myeloid leukemia cell differentiation protein Mcl-1
1-acylglycerol-3-phosphate O-acyltransferase beta	Cytochrome P450 19A1	10 kDa heat shock protein, mitochondrial	Adenosine A1 receptor	Glutathione S-transferase Pi	Heme oxygenase 1	Carboxy-terminal domain RNA polymerase II polypeptide A small phosphatase 1
	MAP kinase-activated protein kinase 2	60 kDa heat shock protein, mitochondrial	Serine/threonine-protein kinase Aurora-B	Histone deacetylase 4	Eukaryotic initiation factor 4A-I	Apoptotic protease-activating factor 1
	Carnitine O-palmitoyltransferase 1 liver isoform	Multidrug resistance-associated protein 4	Calcium sensing receptor	Neprilysin	72 kDa type IV collagenase	Tumor necrosis factor

**Table 7:** Physicochemical and pharmacokinetic properties of the studied ligands.

**ID**	**MW**	**accptHB**	**donorHB**	**QPlogPo/w**	**Rule** **OfFive**	**PSA**	**#Rotor**	**CIQP** **logS**
**2b**	410.935	4	1	6.005	1	41.544	5	−7.236
**4d**	478.934	4	1	6.986	1	41.011	5	−8.646
**4e**	455.933	5	1	5.283	1	85.806	6	−7.758
**4f**	467.987	6.5	2	4.932	0	85.243	6	−7.325
**4h**	445.38	4	1	6.535	1	38.018	5	−7.949
**4i**	428.926	4	1	6.201	1	42.618	5	−7.608
**4j**	478.934	4	1	6.949	1	45.48	5	−8.646
**4k**	455.933	5	1	5.082	1	87.682	6	−7.758
**4l**	467.987	6.5	2	5.289	1	86.742	6	−7.325
**4m**	479.825	4	1	6.96	1	37.855	5	−8.665
**ID**	**Percent** **Human** **Oral** **Absorption**	**QPPCaco**	**QPPMDCK**	**QPlogBB**	**QPlog** **HERG**	**QPlogKhsa**	**CNS**	**#metab**
**2b**	100	4797.201	10,000	0.264	−7.033	0.884	1	4
**4d**	100	5598.932	10,000	0.616	−6.735	1.136	2	3
**4e**	95.015	629.287	1753.816	−0.773	−6.819	0.818	−1	4
**4f**	100	675.963	1383.764	−0.86	−7.087	0.69	−1	3
**4h**	100	5564.24	10,000	0.509	−7.021	0.991	2	4
**4i**	100	4890.181	10,000	0.387	−6.754	0.916	1	4
**4j**	100	3372.682	10,000	0.324	−7.26	1.185	1	5
**4k**	90.944	433.678	857.42	−1.064	−7.137	0.81	−2	5
**4l**	100	896.897	2297.395	−0.712	−7.352	0.763	−1	5
**4m**	100	5649.57	10,000	0.654	−6.934	1.096	2	3

QPlogPo/w: Predicted octanol/water partition coefficient (−2.0–6.5); RuleOfFive: Number of violations of Lipinski’s rule of five; PSA: Van der Waals surface area of polar nitrogen and oxygen atoms and carbonyl carbon atoms (7–200); #rotor: Number of non-trivial (not CX3), non-hindered (not alkene, amide, small ring) rotatable bonds. (0–15); CIQPlogS: Conformation Independent predicted aqueous solubility, log S. S in mol dm^−3^ is the concentration of the solute in a saturated solution (−6.5–0.5); PercentHumanOralAbsorption: Predicted human oral absorption on a 0 to 100% scale; QPPCaco: Predicted apparent Caco-2 cell permeability in nm/sec. Caco-2 cells are a model for the gut–blood barrier (<25 poor, >500 great); QPPMDCK: Predicted apparent MDCK cell permeability in nm/sec. MDCK cells are considered to be a good mimic for the blood–brain barrier (<25 poor, >500 great); QPlogBB: Predicted brain/blood partition coefficient (−3.0–1.2); QPlogHERG: Predicted IC50 value for the blockage of HERG K+ channels (concern below −5); QPlogKhsa: Prediction of binding to human serum albumin (−1.5–1.5); CNS: Predicted central nervous system activity on a −2 (inactive) to +2 (active) scale; #metab: Number of likely metabolic reactions.

## Data Availability

Data is contained within the article and Supplementary Material.

## Supplementary Materials

The following supporting information can be downloaded at: https://www.mdpi.com/article/10.3390/ph15080937/s1.
