# Supplementary material for: Benzothiazole Derivatives Endowed with Antiproliferative Activity in Paraganglioma and Pancreatic Cancer Cells: Structure–Activity Relationship Studies and Target Prediction Analysis
Source: Pharmaceuticals (Basel). 2022 Jul 28;15(8):937. doi: 10.3390/ph15080937 (PMC9412555; doi:10.3390/ph15080937)

Supporting Information

Benzothiazole Derivatives Endowed with Antiproliferative Activity in Paraganglioma and Pancreatic Cancer Cells: Structure-Activity Relationship Studies and Target Prediction Analysis.

Rosa Amoroso[1,#], Laura De Lellis[1,2,#], Rosalba Florio[1], Nazaret Moreno Rodriguez[3], Mariangela Agamennone[1], Barbara De Filippis[1], Letizia Giampietro[1], Cristina Maccallini[1], Inmaculada Fernández[3], Rocío Recio[3], Alessandro Cama[1,2], Marialuigia Fantacuzzi[1,*], Alessandra Ammazzalorso[1,*]

1 Department of Pharmacy, "G. d'Annunzio" University of Chieti-Pescara, Via Dei Vestini 31, 66100 Chieti, Italy; rosa.amoroso@unich.it (R.A.); laura.delellis@unich.it (L.D.L.); rosalba.florio@unich.it (R.F.); mariangela.agamennone@unich.it (M.A.); barbara.defilippis@unich.it (B.D.F.); letizia.giampietro@unich.it (L.G.); cristina.maccallini@unich.it (C.M.); alessandro.cama@unich.it (A.C.).

2 Center for Advanced Studies and Technology CAST, Via Luigi Polacchi 11; 66100 Chieti, Italy.

3 Departamento de Química Orgánica y Farmacéutica, Facultad de Farmacia, Universidad de Sevilla, C/Profesor García González, 2, 41012, Sevilla, Spain; nmoreno4@us.es (N.M.R.); inmaff@us.es (I.F.F.); rrecioj@us.es (R. R.).

# These authors contributed equally to the work

Correspondence: alessandra.ammazzalorso@unich.it (A.A.); marialuigia.fantacuzzi@unich.it (M.F.).

*Corresponding authors: Dr. Alessandra Ammazzalorso, Department of Pharmacy, "G. d'Annunzio" University, Via dei Vestini 31, 66100 Chieti, Italy; Ph. +39 0871 3554682; alessandra.ammazzalorso@unich.it;
Dr. Marialuigia Fantacuzzi, Department of Pharmacy, "G. d'Annunzio" University, Via dei Vestini 31, 66100 Chieti, Italy; Ph. +39 0871 3554684; marialuigia.fantacuzzi@unich.it.

Table of Contents:

Supplementary Table S1………………………………………………………….. page 2
Supplementary Figure S1…………………………………………………………page 3
Supplementary Figure S2………………………………………………………….. page 4
Supplementary Figure S3………………………………………………………….. page 4
^{1}H and ^{13}C spectra of final compounds **4a-p**……………………………………………… page 5

Supplementary Table S1. Selectivity index (SI) values for compounds **2b**, **4d**, **4e**, **4f**, **4h**, **4i**, **4j** and **4m**. SI values were calculated for each compound as follows: IC_{50} in normal (HFF-1) fibroblast cells/IC_{50} in pancreatic (AsPC-1, BxPC-3 and Capan-2), or paraganglioma (PTJ64i, PTJ86i) cancer cells (see IC_{50} values in **Table 2** of the main text).

	**Selectivity index (SI) values**				
	**Pancreatic cancer**			**Paraganglioma**	
	**AsPC-1**	**BxPC-3**	**Capan-2**	**PTJ64i**	**PTJ86i**
**2b**	1.72	1.43	1.09	2.52	1.28
**4d**	1.20	2.31	1.03	1.36	0.74
**4e**	1.31	1.56	1.18	1.70	0.88
**4f**	0.65	0.35	0.33	0.53	0.51
**4h**	0.95	0.96	0.65	1.59	0.70
**4i**	1.01	0.81	0.53	1.74	1.28
**4j**	1.90	1.30	0.75	1.62	1.04
**4m**	1.22	1.05	0.77	1.32	0.52

Supplementary Figure S1. Effect of combined treatments with compound **4k** and gemcitabine on the viability of pancreatic cancer (AsPC-1, BxPC-3 and Capan-2) and normal (HFF-1) fibroblast cells. Cell viability was assessed by MTT assays after a 72-h incubation of cells with **4k** and gemcitabine at the indicated concentrations, as single agents or in combination. Histograms represent the means ±SD of two independent experiments with quintuplicate determinations. Combination indexes (CIs) were calculated by CompuSyn software. Combinations assessed as synergistic by CIs < 1 are indicated.

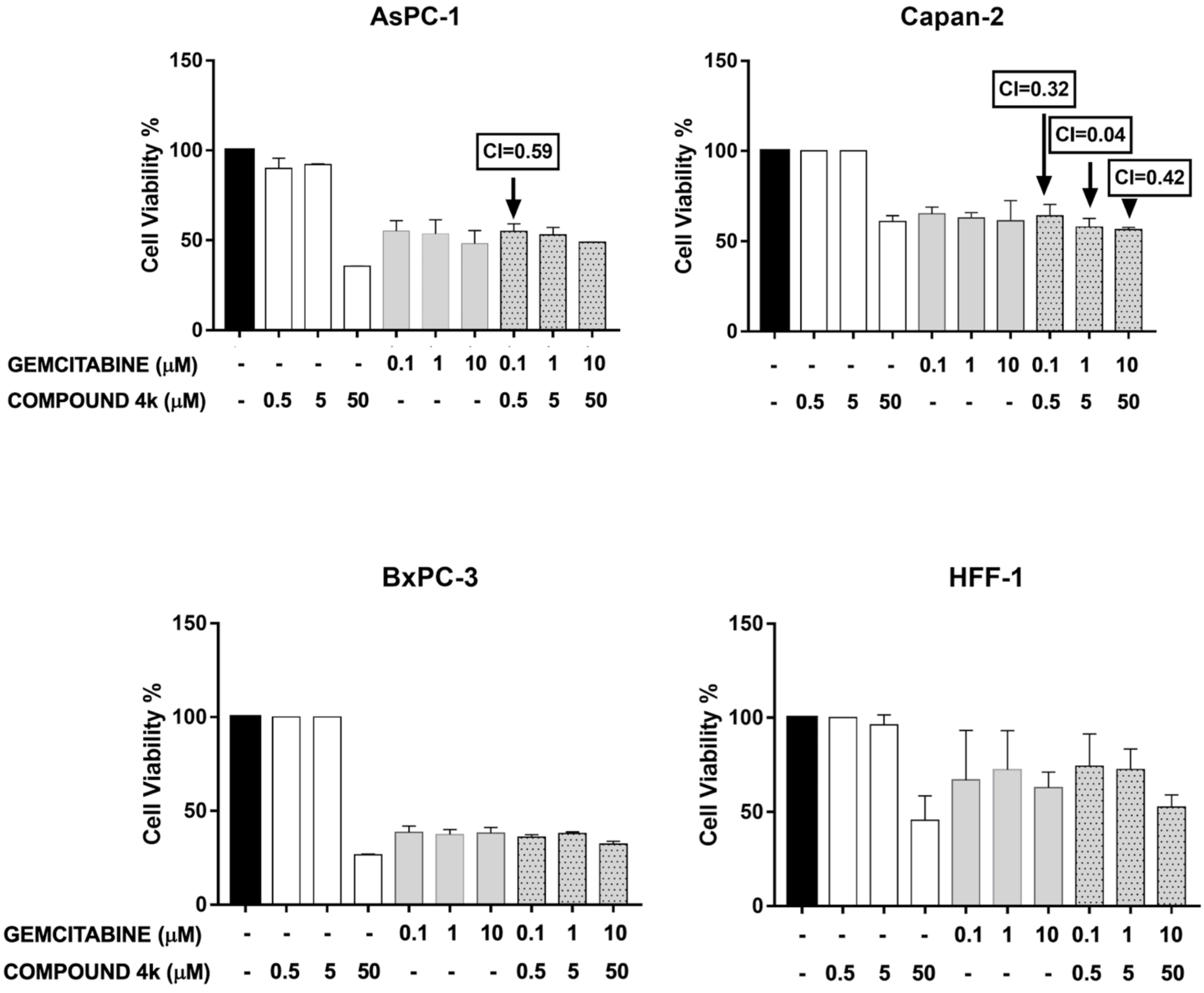

Supplementary Figure S2. Interaction diagram of compounds **2b**, **4a-p** in the catalytic site of CBR1 (PDB:6KPG).

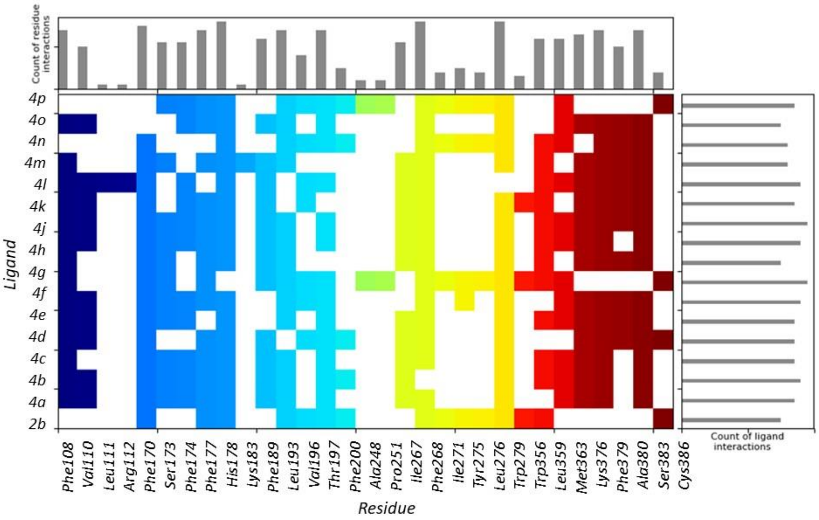

Supplementary Figure S3. Interaction diagram of compounds **2b**, **4a-p** in the catalytic site of the chimera SENP2-loop1 of SENP6 (PDB:3ZO5).

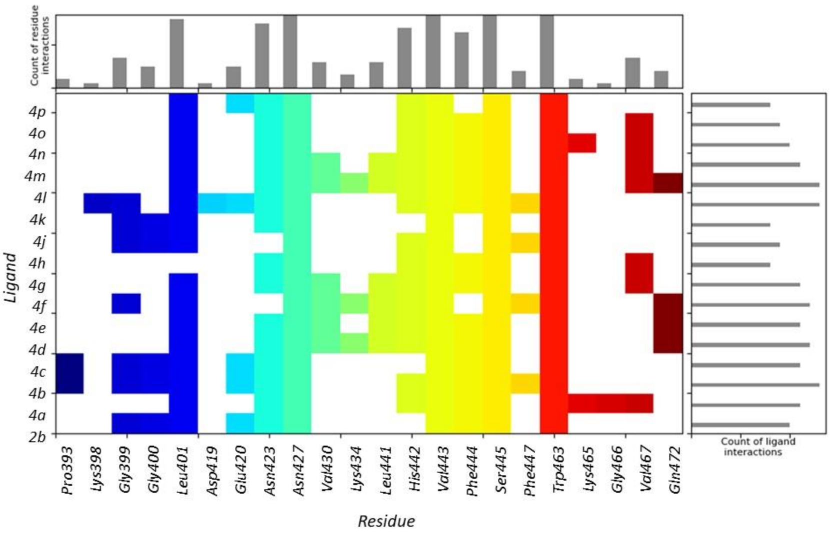

Gradient Shimming

Sample Name:
AA943A
Data Collected on:
m300-mercury300
Archive directory:
/home/ammazza/vnmrsys/data
Sample directory:
MF255_20150422_01
FidFile: AA943A

Pulse Sequence: PROTON (s2pul)
Solvent: cdcl3
Data collected on: Jul 9 2020

Operator: ammazza

Relax. delay 1.000 sec
Pulse 45.0 degrees
Acq. time 1.706 sec
Width 4803.1 Hz
16 repetitions
OBSERVE H1, 300.1976543 MHz
DATA PROCESSING
FT size 16384
Total time 0 min 45 sec

4a

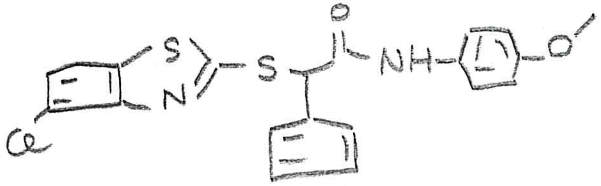

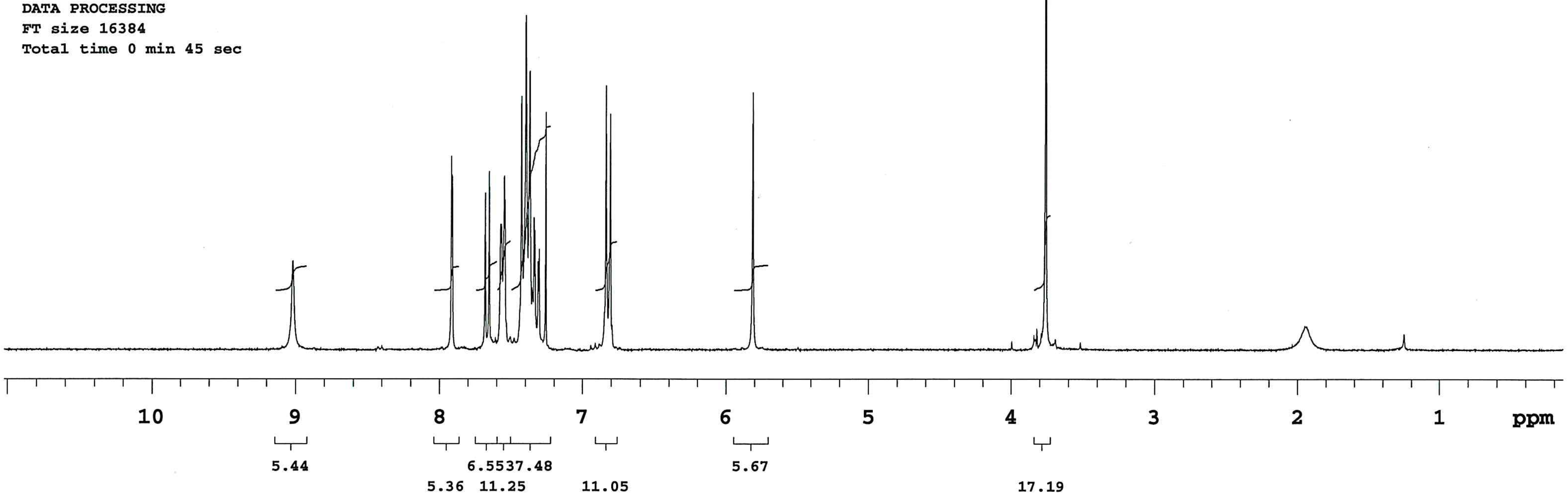

4a

Gradient Shimming

Sample Name:
AA943A
Data Collected on:
m300-mercury300
Archive directory:
/home/ammazza/vnmrsys/data
Sample directory:
MF255_20150422_01
FidFile: CARBON

Pulse Sequence: CARBON (s2pul)
Solvent: cdcl3
Data collected on: Jul 9 2020

Temp. 20.5 C / 293.6 K
Operator: ammazza

Relax. delay 1.000 sec
Pulse 45.0 degrees
Acq. time 0.868 sec
Width 18867.9 Hz
2880 repetitions
OBSERVE C13, 75.4847602 MHz
DECOUPLE H1, 300.1991980 MHz
Power 38 dB
continuously on
WALTZ-16 modulated
DATA PROCESSING
Line broadening 0.5 Hz
FT size 32768
Total time 21 hr, 28 min

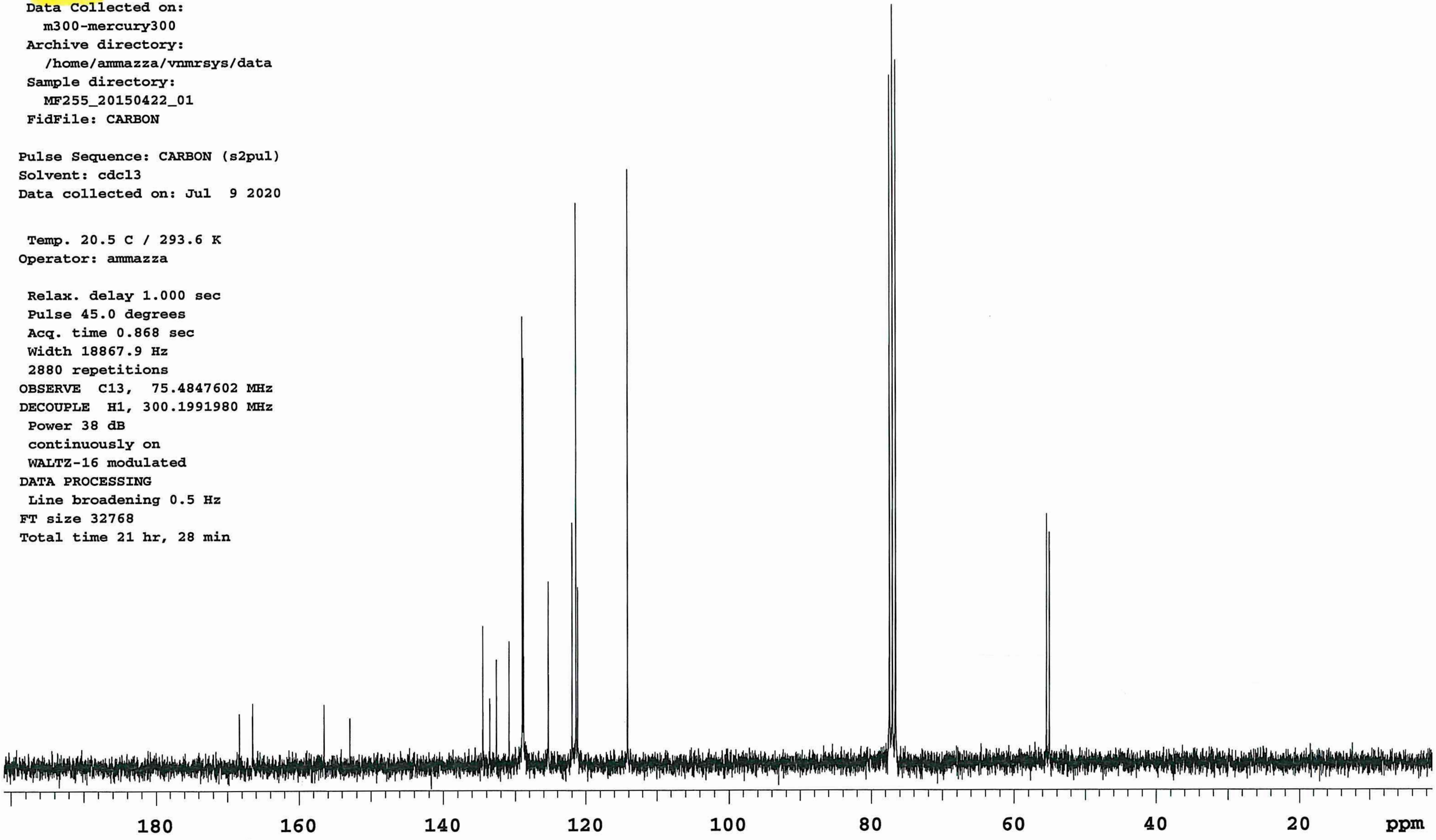

4b

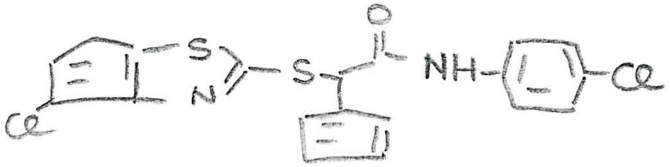

Gradient Shimming

Sample Name:
AA949B
Data Collected on:
m300-mercury300
Archive directory:
/home/ammazza/vnmrsys/data
Sample directory:
MF255_20150422_01
FidFile: PROTON

Pulse Sequence: PROTON (s2pul)
Solvent: cdcl3
Data collected on: Sep 28 2020

Operator: ammazza

Relax. delay 1.000 sec
Pulse 45.0 degrees
Acq. time 1.706 sec
Width 4803.1 Hz
16 repetitions
OBSERVE H1, 300.1976543 MHz
DATA PROCESSING
FT size 16384
Total time 0 min 45 sec

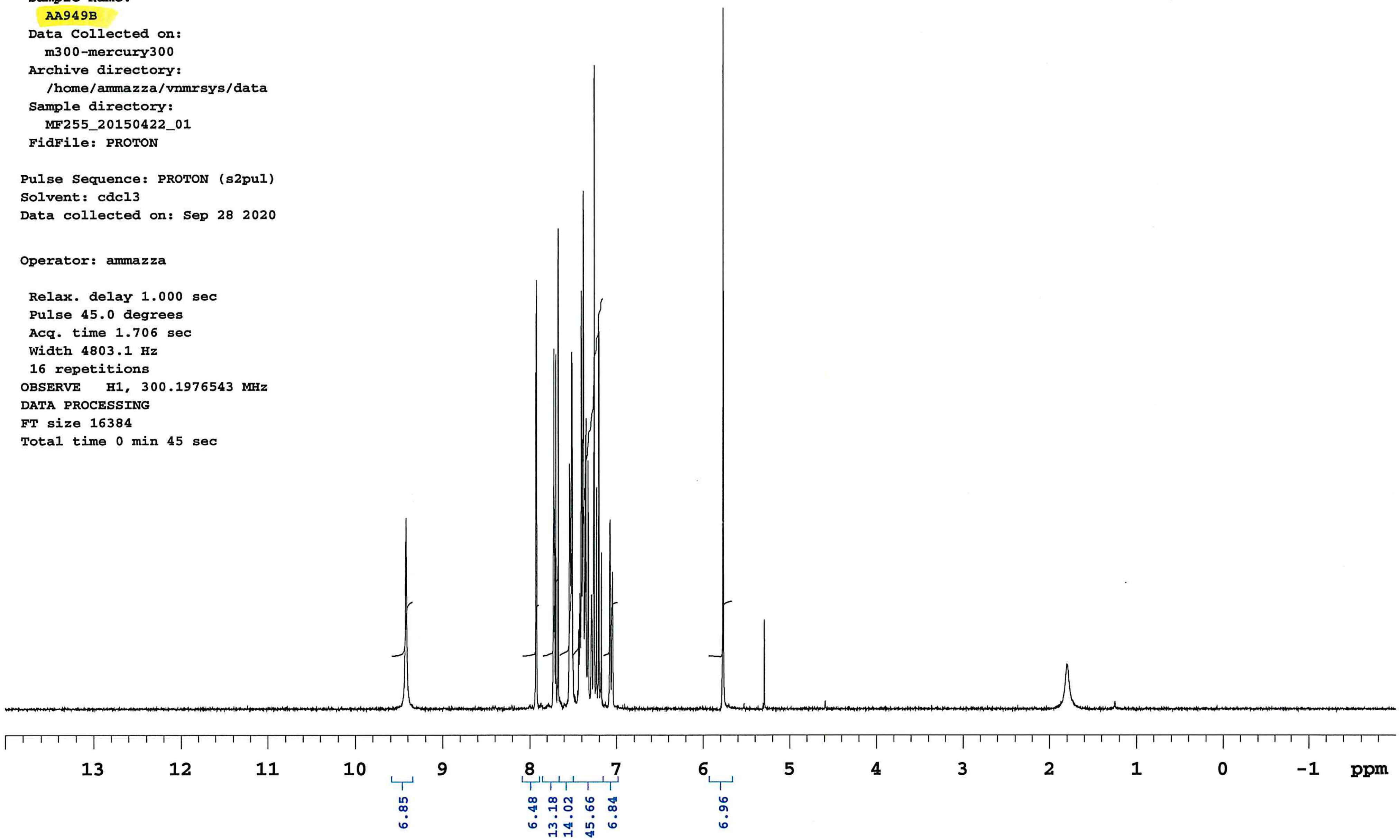

4b

Gradient Shimming

Sample Name:
AA948A
Data Collected on:
m300-mercury300
Archive directory:
/home/ammazza/vnmrsys/data
Sample directory:
MF255_20150422_01
FidFile: AA948A_C13

Pulse Sequence: CARBON (s2pul)
Solvent: cdcl3
Data collected on: Sep 28 2020

Operator: ammazza

Relax. delay 1.000 sec
Pulse 45.0 degrees
Acq. time 0.868 sec
Width 18867.9 Hz
1080 repetitions
OBSERVE C13, 75.4847602 MHz
DECOUPLE H1, 300.1991980 MHz
Power 38 dB
continuously on
WALTZ-16 modulated
DATA PROCESSING
Line broadening 0.5 Hz
FT size 32768
Total time 16 hr, 6 min

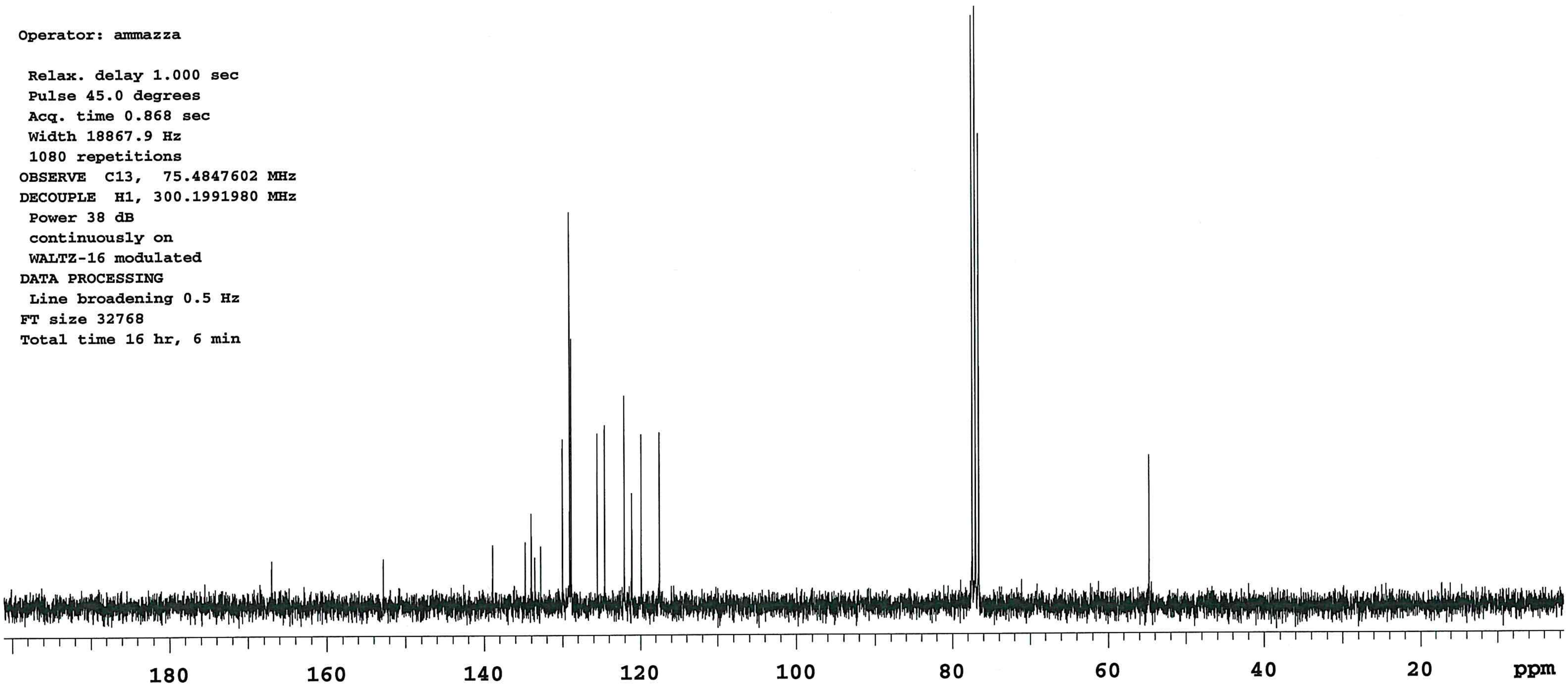

4C

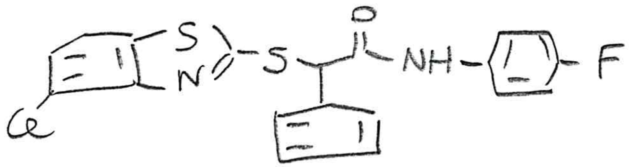

Gradient Shimming

Sample Name:
AA701A
Data Collected on:
m300-mercury300
Archive directory:
/export/home/chempack/vnmrsys/data
Sample directory:

FidFile: AA701A

Pulse Sequence: PROTON (s2pul)
Solvent: cdcl3
Data collected on: Nov 19 2015

Temp. 18.0 C / 291.1 K
Operator: ammazza

Relax. delay 1.000 sec
Pulse 45.0 degrees
Acq. time 1.706 sec
Width 4803.1 Hz
16 repetitions
OBSERVE H1, 300.1976543 MHz
DATA PROCESSING
FT size 16384
Total time 0 min 45 sec

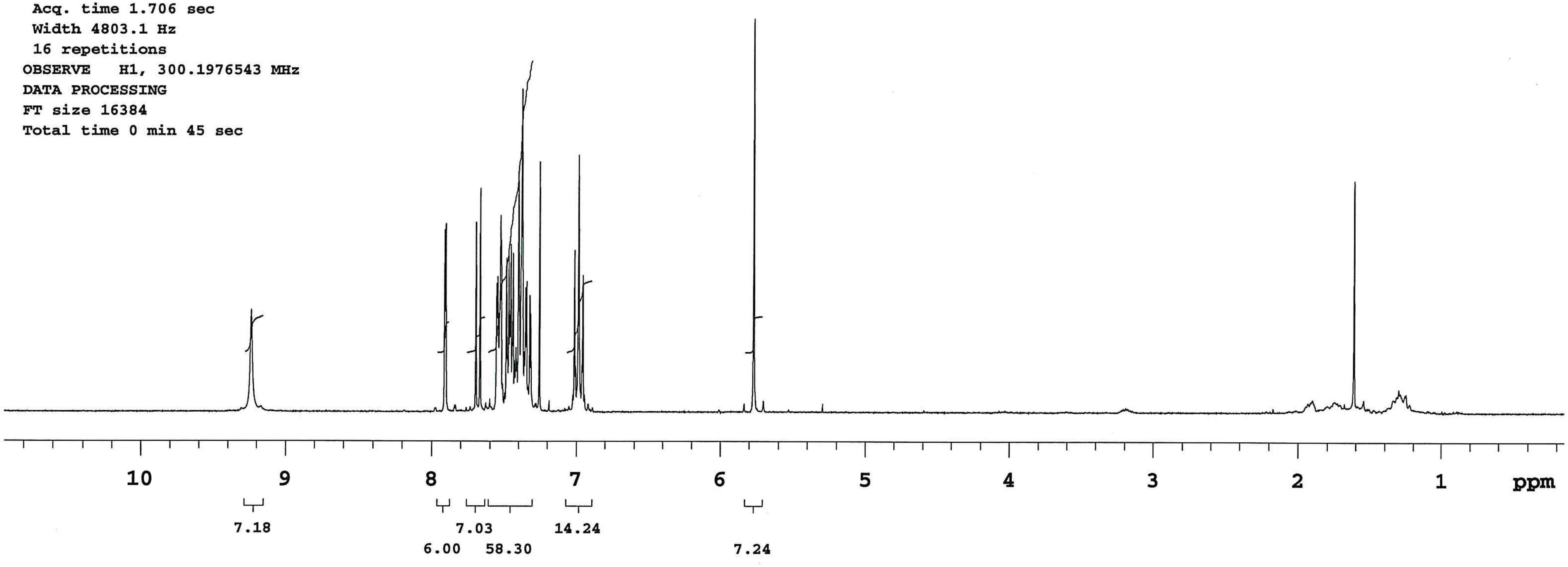

4c

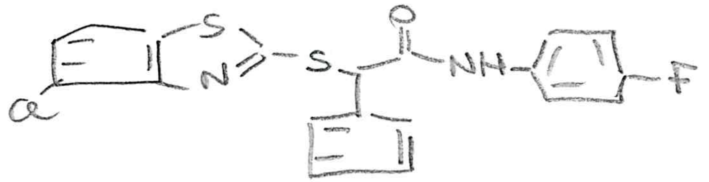

Gradient Shimming

Sample Name:
AA701A
Data Collected on:
m300-mercury300
Archive directory:
/export/home/chempack/vnmrsys/data
Sample directory:

FidFile: CARBON

Pulse Sequence: CARBON (s2pul)
Solvent: cdcl3
Data collected on: Nov 19 2015

Temp. 20.7 C / 293.9 K
Operator: ammazza

Relax. delay 1.000 sec
Pulse 45.0 degrees
Acq. time 0.868 sec
Width 18867.9 Hz
640 repetitions
OBSERVE C13, 75.4847602 MHz
DECOUPLE H1, 300.1991980 MHz
Power 38 dB
continuously on
WALTZ-16 modulated
DATA PROCESSING
Line broadening 0.5 Hz
FT size 32768
Total time 1 hr, 36 min

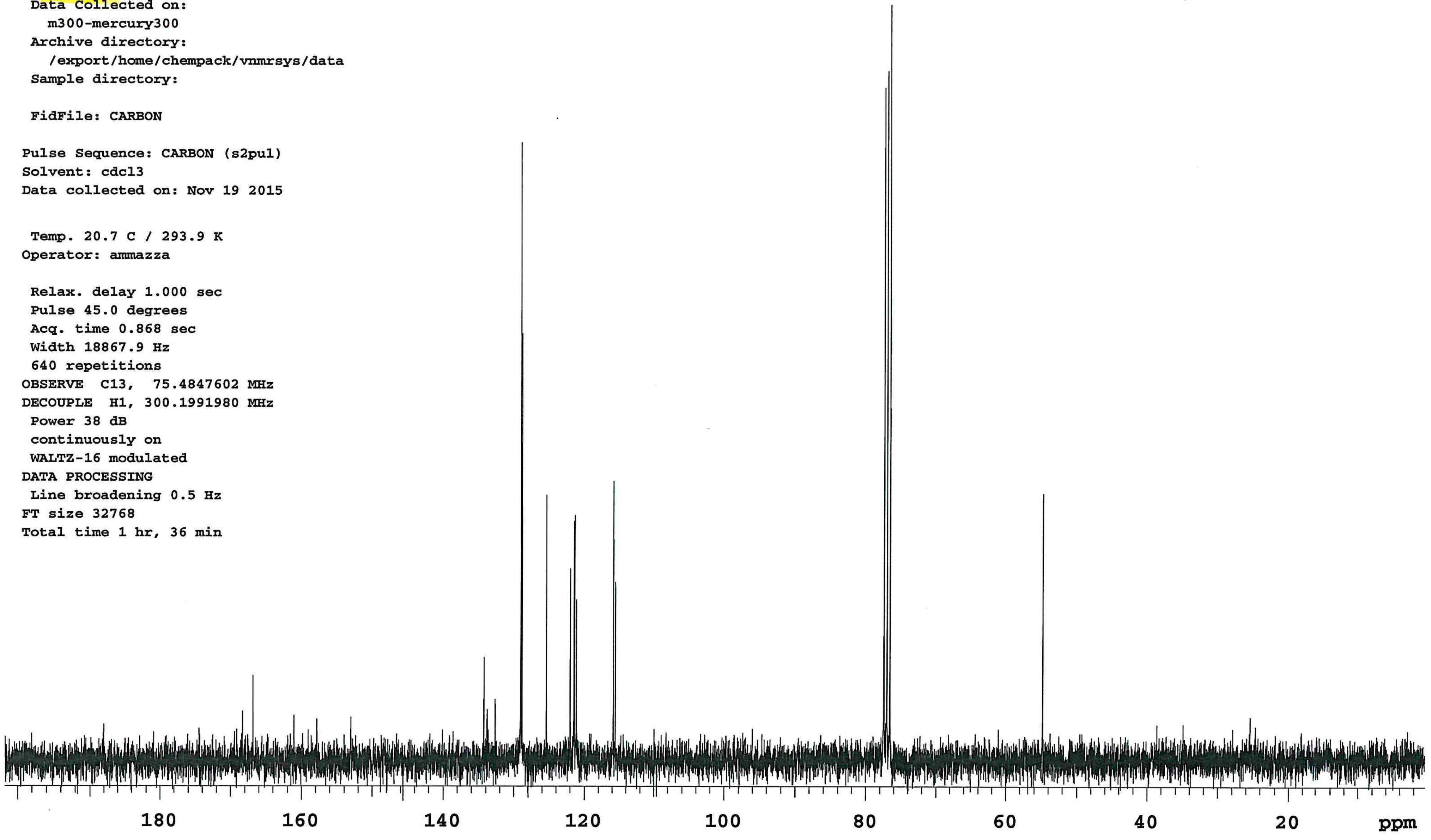

Gradient Shimming

Sample Name:
AA955A
Data Collected on:
m300-mercury300
Archive directory:
/home/ammazza/vnmrsys/data
Sample directory:
MF255_20150422_01
FidFile: AA955A

Pulse Sequence: PROTON (s2pul)
Solvent: cdcl3
Data collected on: Nov 11 2020

Operator: ammazza

Relax. delay 1.000 sec
Pulse 45.0 degrees
Acq. time 1.706 sec
Width 4803.1 Hz
16 repetitions
OBSERVE H1, 300.1976543 MHz
DATA PROCESSING
FT size 16384
Total time 0 min 45 sec

4d

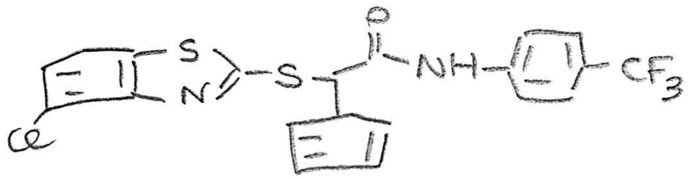

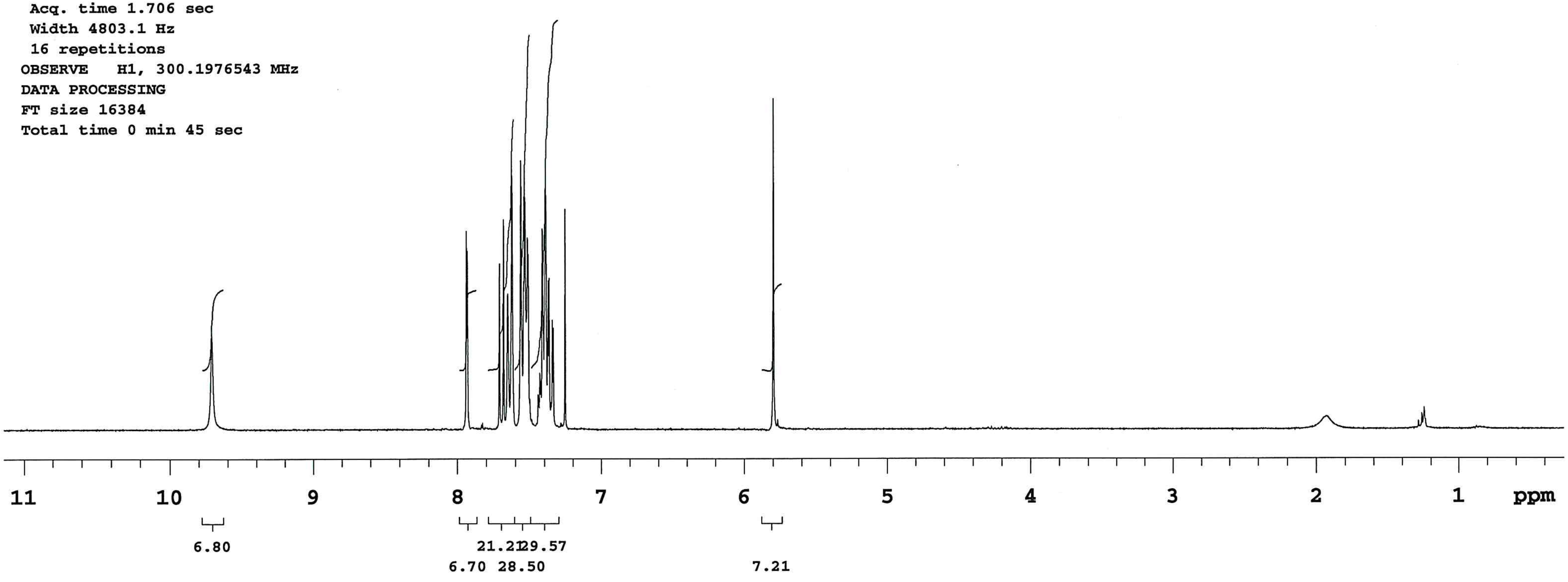

4d

Gradient Shimming

Sample Name:
AA955A
Data Collected on:
m300-mercury300
Archive directory:
/home/ammazza/vnmrsys/data
Sample directory:
MF255_20150422_01
FidFile: CARBON

Pulse Sequence: CARBON (s2pul)
Solvent: cdcl3
Data collected on: Nov 11 2020

Temp. 17.6 C / 290.8 K
Operator: ammazza

Relax. delay 1.000 sec
Pulse 45.0 degrees
Acq. time 0.868 sec
Width 18867.9 Hz
2688 repetitions
OBSERVE C13, 75.4847602 MHz
DECOUPLE H1, 300.1991980 MHz
Power 38 dB
continuously on
WALTZ-16 modulated
DATA PROCESSING
Line broadening 0.5 Hz
FT size 32768
Total time 2 hr, 8 min

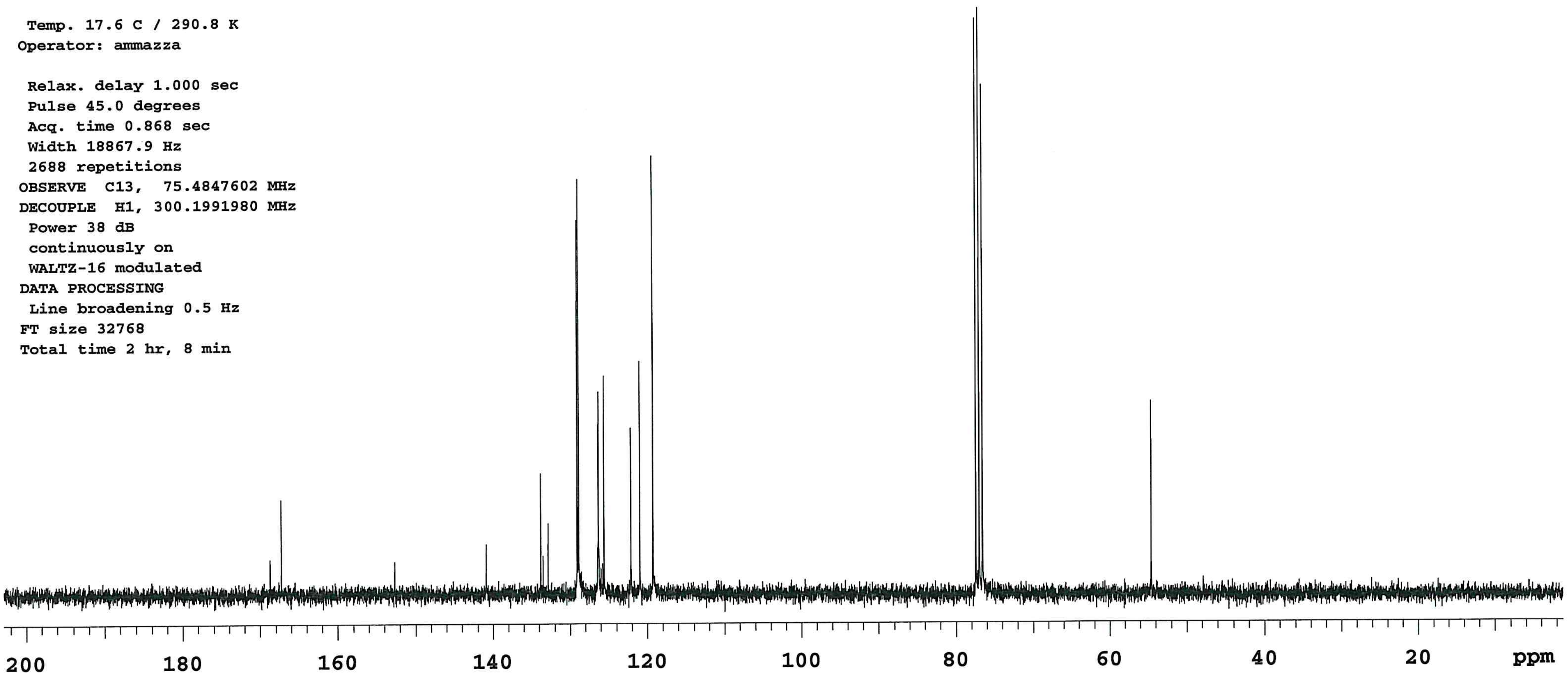

4e

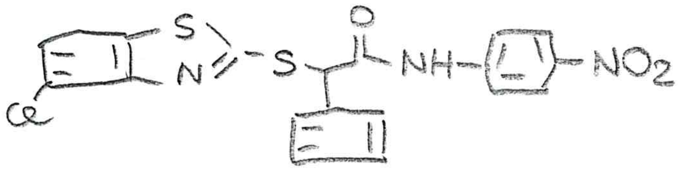

Gradient Shimming

Sample Name:
AA952A
Data Collected on:
m300-mercury300
Archive directory:
/home/ammazza/vnmrsys/data
Sample directory:
MF255_20150422_01
FidFile: AA952A

Pulse Sequence: PROTON (s2pul)
Solvent: cdcl3
Data collected on: Oct 7 2020

Operator: ammazza

Relax. delay 1.000 sec
Pulse 45.0 degrees
Acq. time 1.706 sec
Width 4803.1 Hz
16 repetitions
OBSERVE H1, 300.1976543 MHz
DATA PROCESSING
FT size 16384
Total time 0 min 45 sec

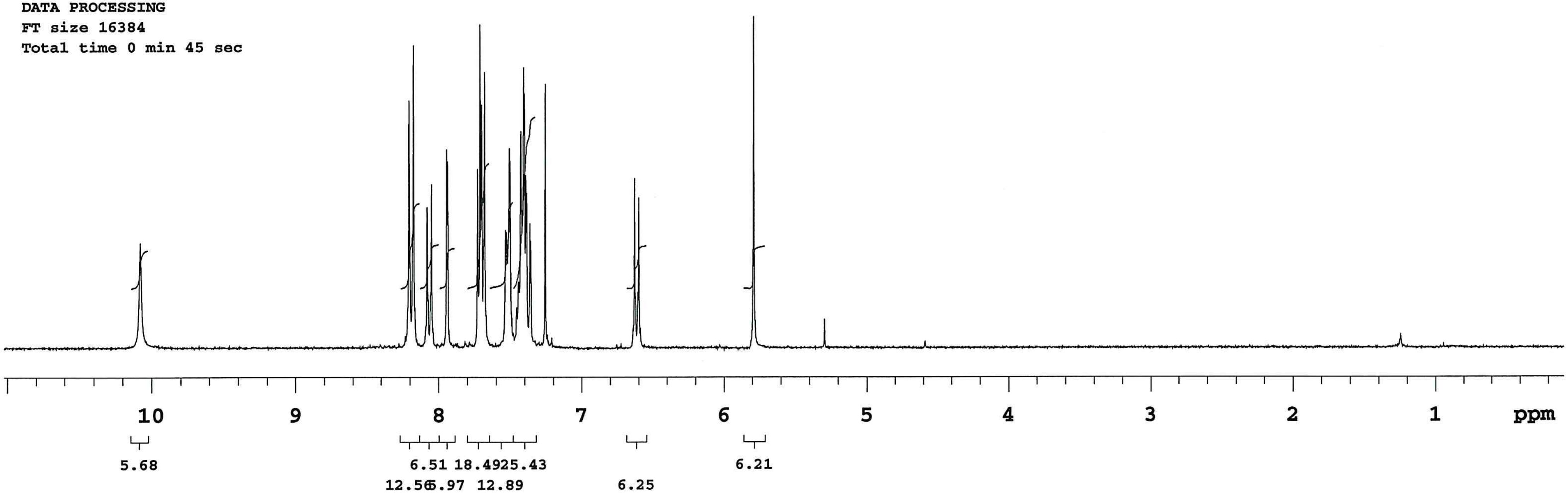

4e

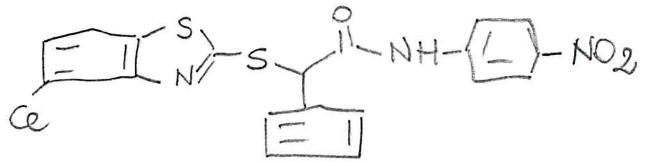

Gradient Shimming

Sample Name:
AA952A
Data Collected on:
m300-mercury300
Archive directory:
/home/ammazza/vnmrsys/data
Sample directory:
MF255_20150422_01
FidFile: CARBON

Pulse Sequence: CARBON (s2pul)
Solvent: cdcl3
Data collected on: Oct 8 2020

Temp. 18.7 C / 291.9 K
Operator: ammazza

Relax. delay 1.000 sec
Pulse 45.0 degrees
Acq. time 0.868 sec
Width 18867.9 Hz
4032 repetitions
OBSERVE C13, 75.4847602 MHz
DECOUPLE H1, 300.1991980 MHz
Power 38 dB
continuously on
WALTZ-16 modulated
DATA PROCESSING
Line broadening 0.5 Hz
FT size 32768
Total time 3 hr, 13 min

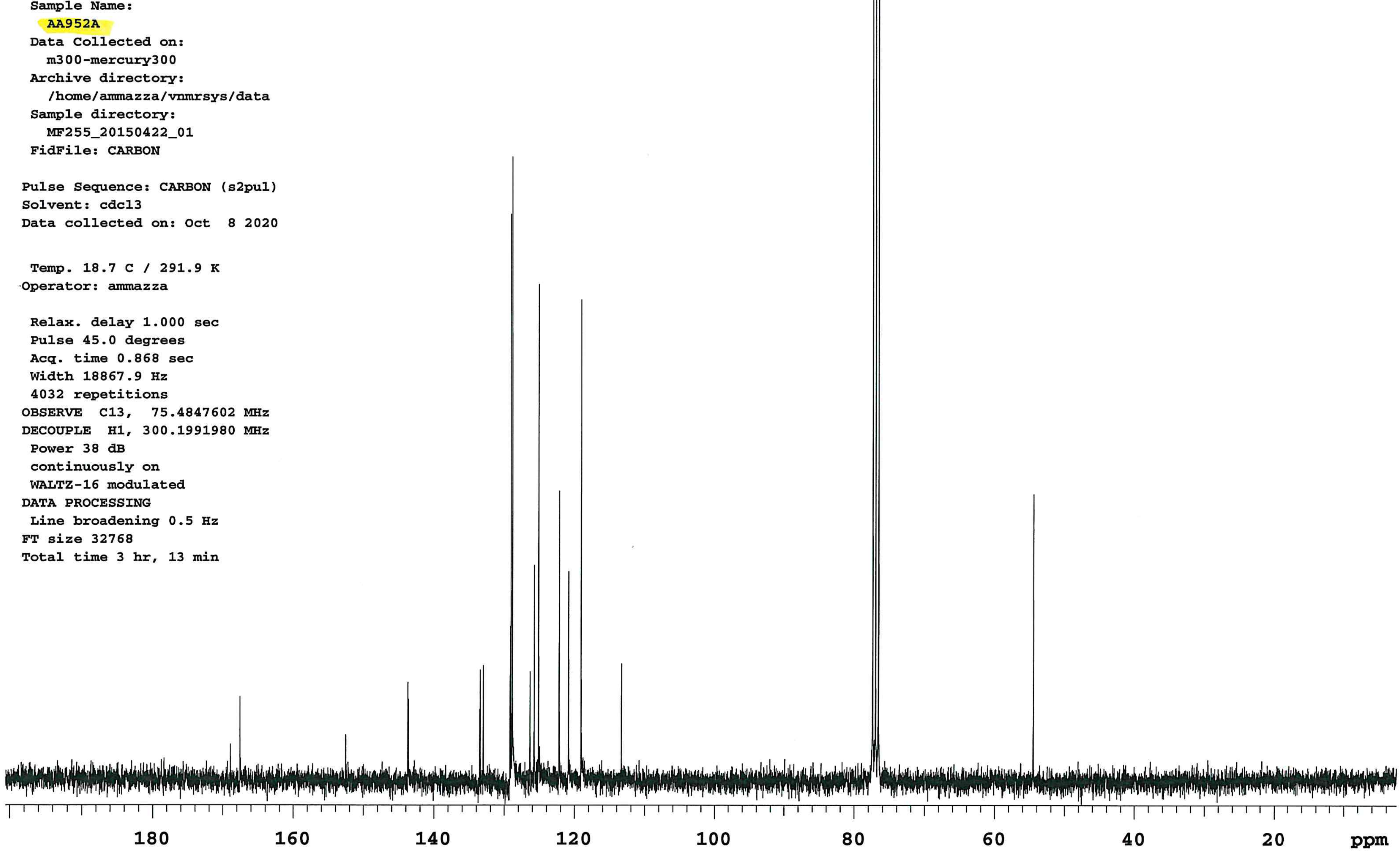

4f

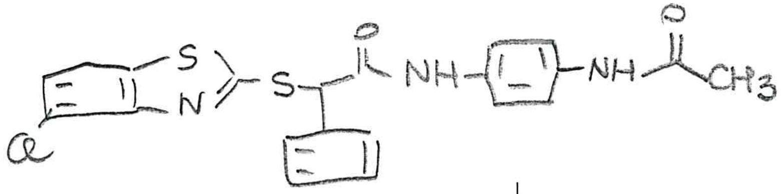

Gradient Shimming

Sample Name:
AA675ppt
Data Collected on:
m300-mercury300
Archive directory:
/export/home/chempack/vnmrsys/data
Sample directory:

FidFile: AA675ppt

Pulse Sequence: PROTON (s2pul)
Solvent: dmso
Data collected on: Oct 27 2014

Operator: ammazza

Relax. delay 1.000 sec
Pulse 45.0 degrees
Acq. time 1.706 sec
Width 4803.1 Hz
16 repetitions
OBSERVE H1, 300.1990802 MHz
DATA PROCESSING
FT size 16384
Total time 0 min 45 sec

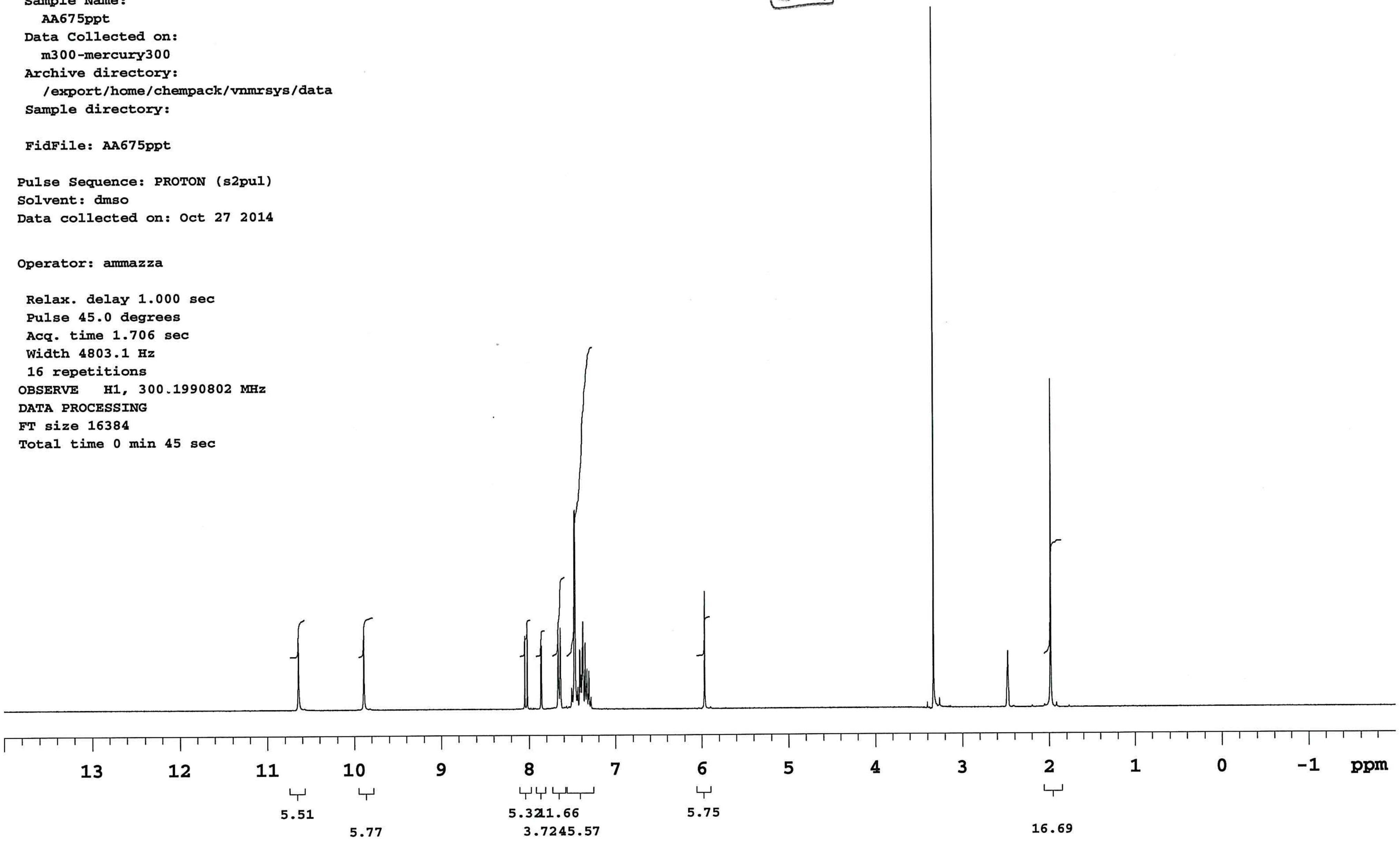

Gradient Shimming

Sample Name:
AA675ppt
Data Collected on:
m300-mercury300
Archive directory:
/export/home/chempack/vnmrsys/data
Sample directory:

FidFile: CARBON

Pulse Sequence: CARBON (s2pul)
Solvent: dmso
Data collected on: Oct 27 2014

Temp. 20.1 C / 293.2 K
Operator: ammazza

Relax. delay 1.000 sec
Pulse 45.0 degrees
Acq. time 0.868 sec
Width 18867.9 Hz
576 repetitions
OBSERVE C13, 75.4851188 MHz
DECOUPLE H1, 300.2006240 MHz
Power 38 dB
continuously on
WALTZ-16 modulated
DATA PROCESSING
Line broadening 0.5 Hz
FT size 32768
Total time 1 hr, 4 min

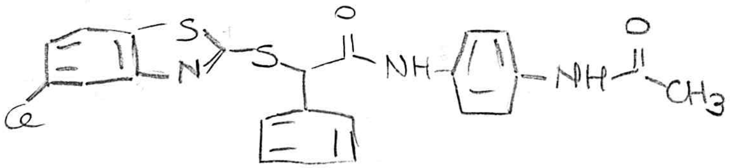

4f

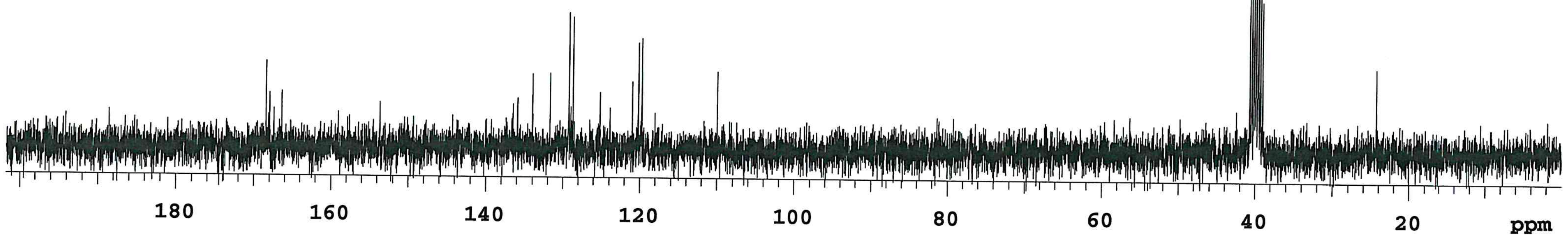

Gradient Shimming

Sample Name:
AA1009A
Data Collected on:
m300-mercury300
Archive directory:
/home/ammazza/vnmrsys/data
Sample directory:
AA1008A_20210409_01
FidFile: AA1009A

Pulse Sequence: PROTON (s2pul)
Solvent: cdcl3
Data collected on: Apr 9 2021

Operator: ammazza

Relax. delay 1.000 sec
Pulse 45.0 degrees
Acq. time 1.706 sec
Width 4803.1 Hz
16 repetitions
OBSERVE H1, 300.1976543 MHz
DATA PROCESSING
FT size 16384
Total time 0 min 45 sec

4g

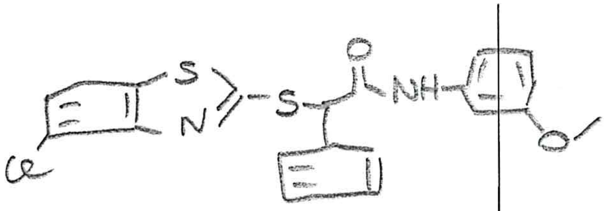

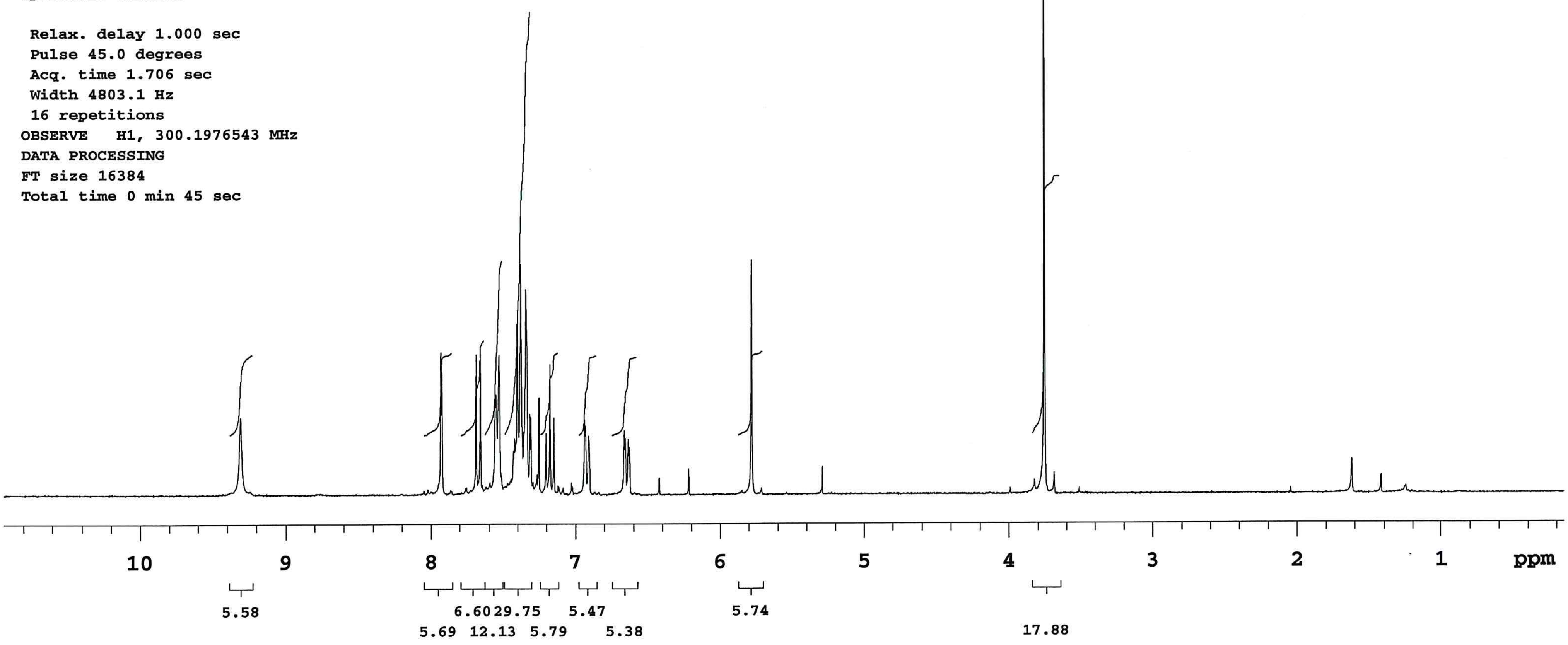

4g

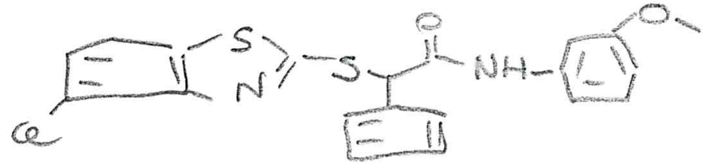

Gradient Shimming

Sample Name:
AA1009A
Data Collected on:
m300-mercury300
Archive directory:
/home/ammazza/vnmrsys/data
Sample directory:
AA1008A_20210409_01
FidFile: CARBON

Pulse Sequence: CARBON (s2pul)
Solvent: cdcl3
Data collected on: Apr 13 2021

Temp. 17.6 C / 290.8 K
Operator: ammazza

Relax. delay 1.000 sec
Pulse 45.0 degrees
Acq. time 0.868 sec
Width 18867.9 Hz
3520 repetitions
OBSERVE C13, 75.4847602 MHz
DECOUPLE H1, 300.1991980 MHz
Power 38 dB
continuously on
WALTZ-16 modulated
DATA PROCESSING
Line broadening 0.5 Hz
FT size 32768
Total time 2 hr, 8 min

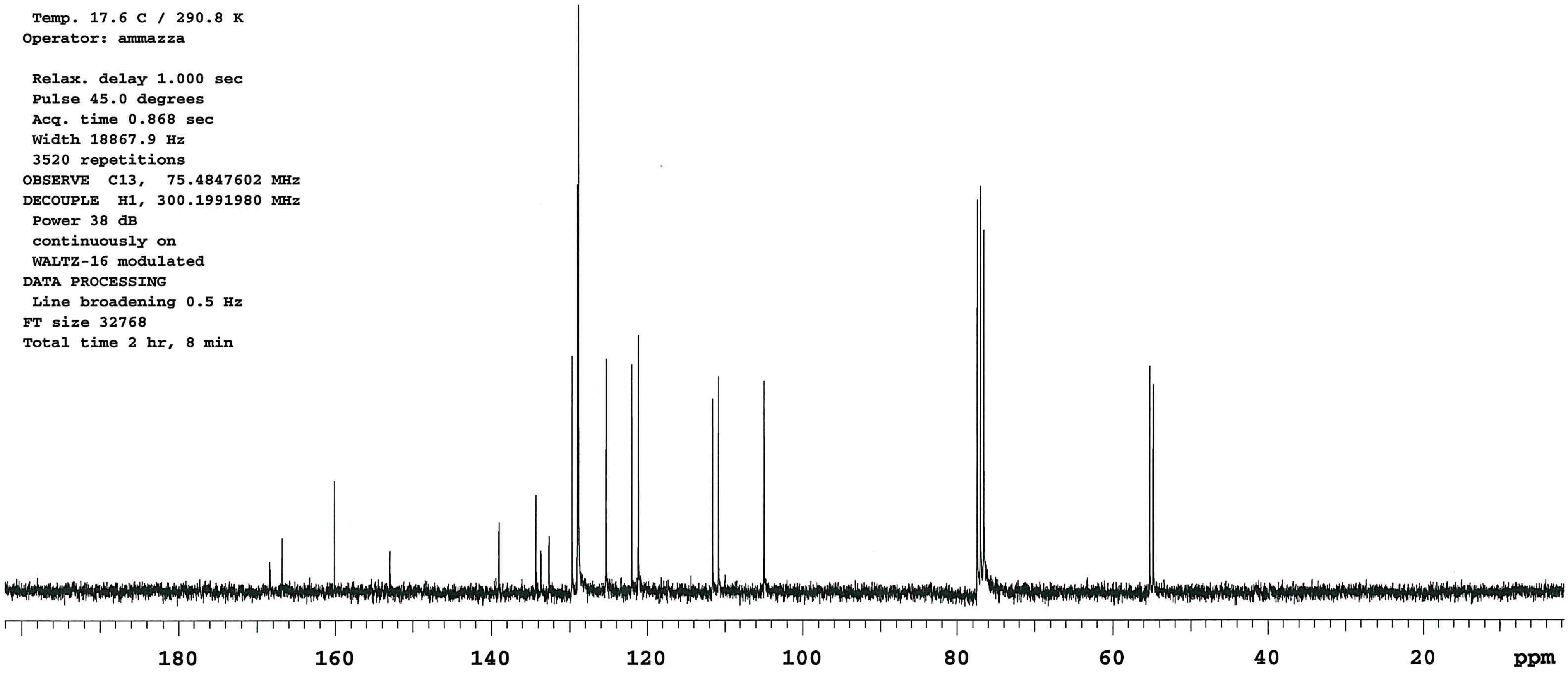

Gradient Shimming

Sample Name:
AA948A
Data Collected on:
m300-mercury300
Archive directory:
/home/ammazza/vnmrsys/data
Sample directory:
MF255_20150422_01
FidFile: AA948A

Pulse Sequence: PROTON (s2pul)
Solvent: cdcl3
Data collected on: Sep 28 2020

Operator: ammazza

Relax. delay 1.000 sec
Pulse 45.0 degrees
Acq. time 1.706 sec
Width 4803.1 Hz
16 repetitions
OBSERVE H1, 300.1976543 MHz
DATA PROCESSING
FT size 16384
Total time 0 min 45 sec

4h

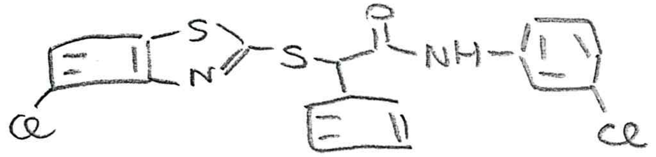

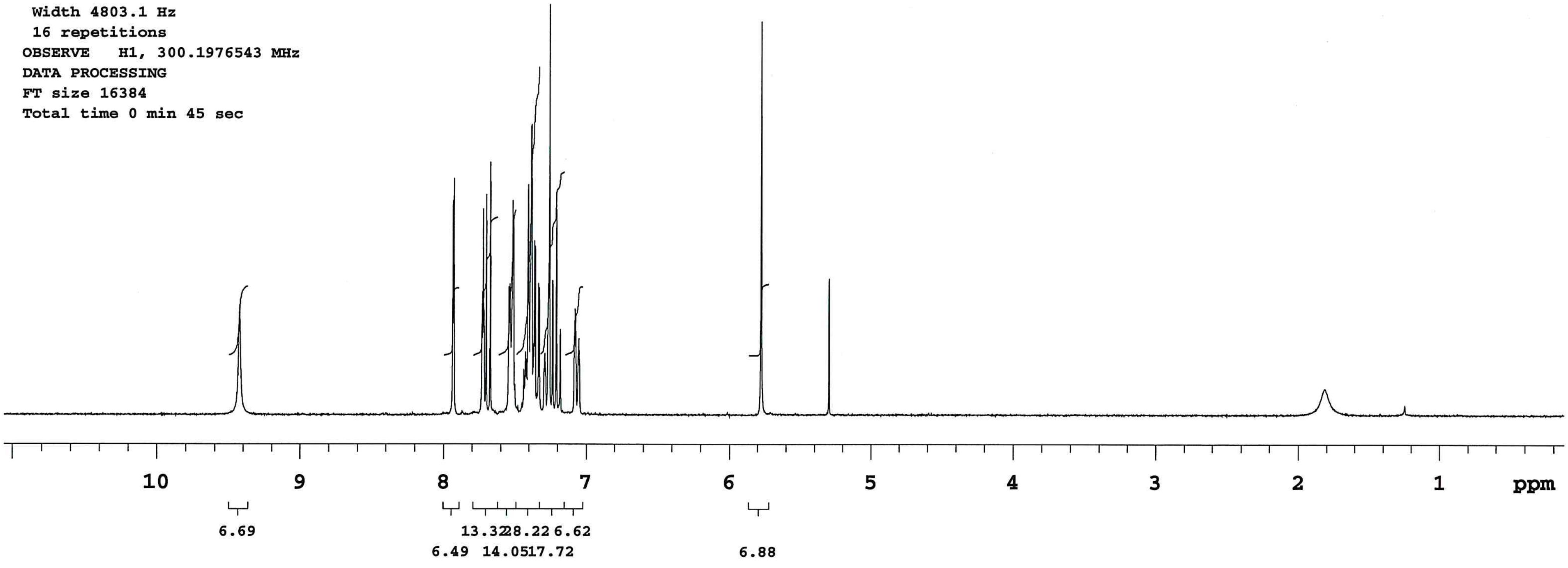

4h

Gradient Shimming

Sample Name:
AA948A
Data Collected on:
m300-mercury300
Archive directory:
/home/ammazza/vnmrsys/data
Sample directory:
MF255_20150422_01
FidFile: CARBON

Pulse Sequence: CARBON (s2pul)
Solvent: cdcl3
Data collected on: Sep 28 2020

Temp. 18.6 C / 291.8 K
Operator: ammazza

Relax. delay 1.000 sec
Pulse 45.0 degrees
Acq. time 0.868 sec
Width 18867.9 Hz
1024 repetitions
OBSERVE C13, 75.4847602 MHz
DECOUPLE H1, 300.1991980 MHz
Power 38 dB
continuously on
WALTZ-16 modulated
DATA PROCESSING
Line broadening 0.5 Hz
FT size 32768
Total time 16 hr, 6 min

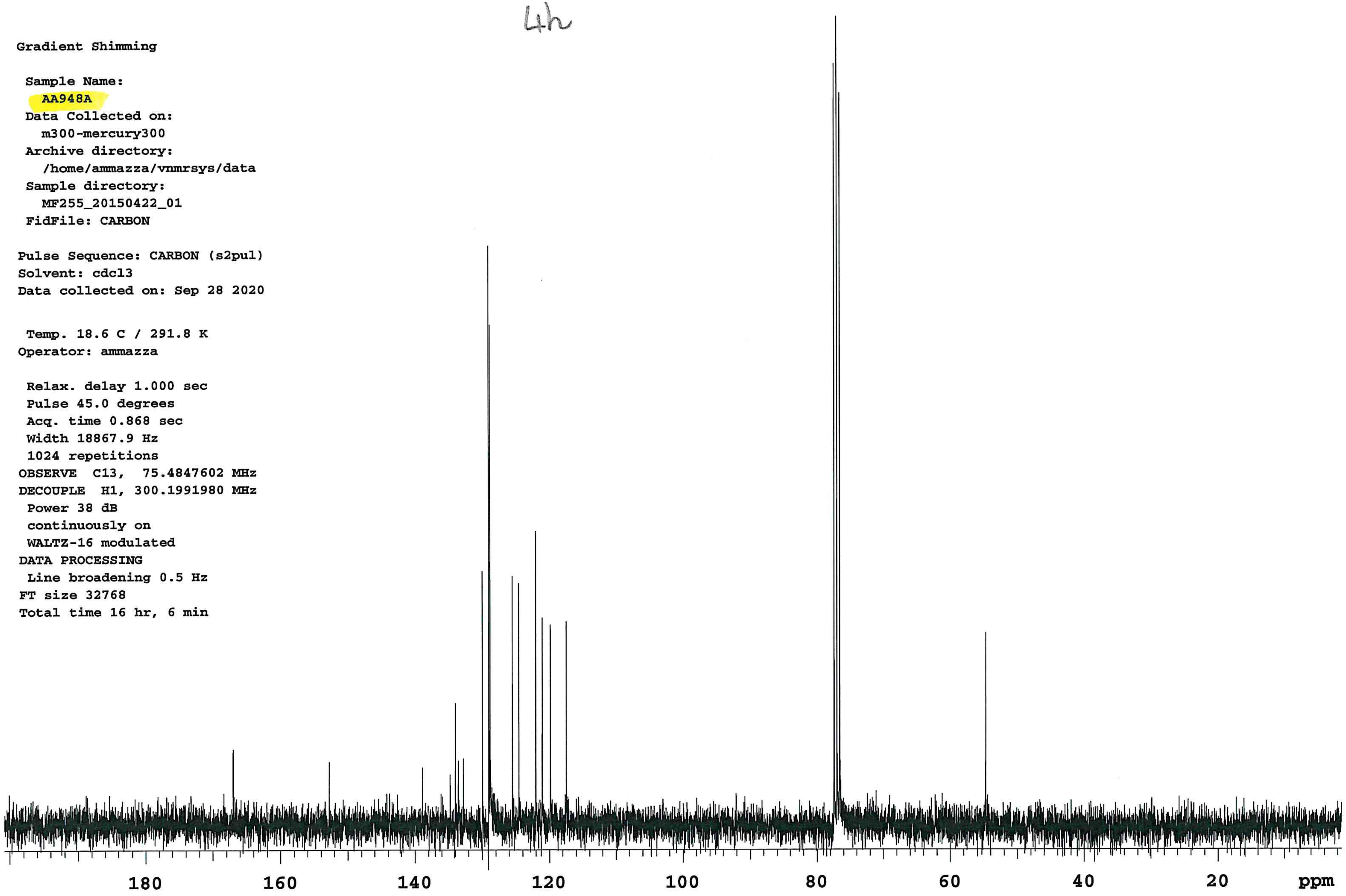

4i

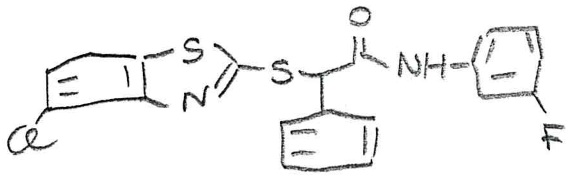

Gradient Shimming

Sample Name:
AA702B
Data Collected on:
m300-mercury300
Archive directory:
/export/home/chempack/vnmrsys/data
Sample directory:

FidFile: AA702B

Pulse Sequence: PROTON (s2pul)
Solvent: cdcl3
Data collected on: Nov 19 2015

Temp. 18.0 C / 291.1 K
Operator: ammazza

Relax. delay 1.000 sec
Pulse 45.0 degrees
Acq. time 1.706 sec
Width 4803.1 Hz
16 repetitions
OBSERVE H1, 300.1976543 MHz
DATA PROCESSING
FT size 16384
Total time 0 min 45 sec

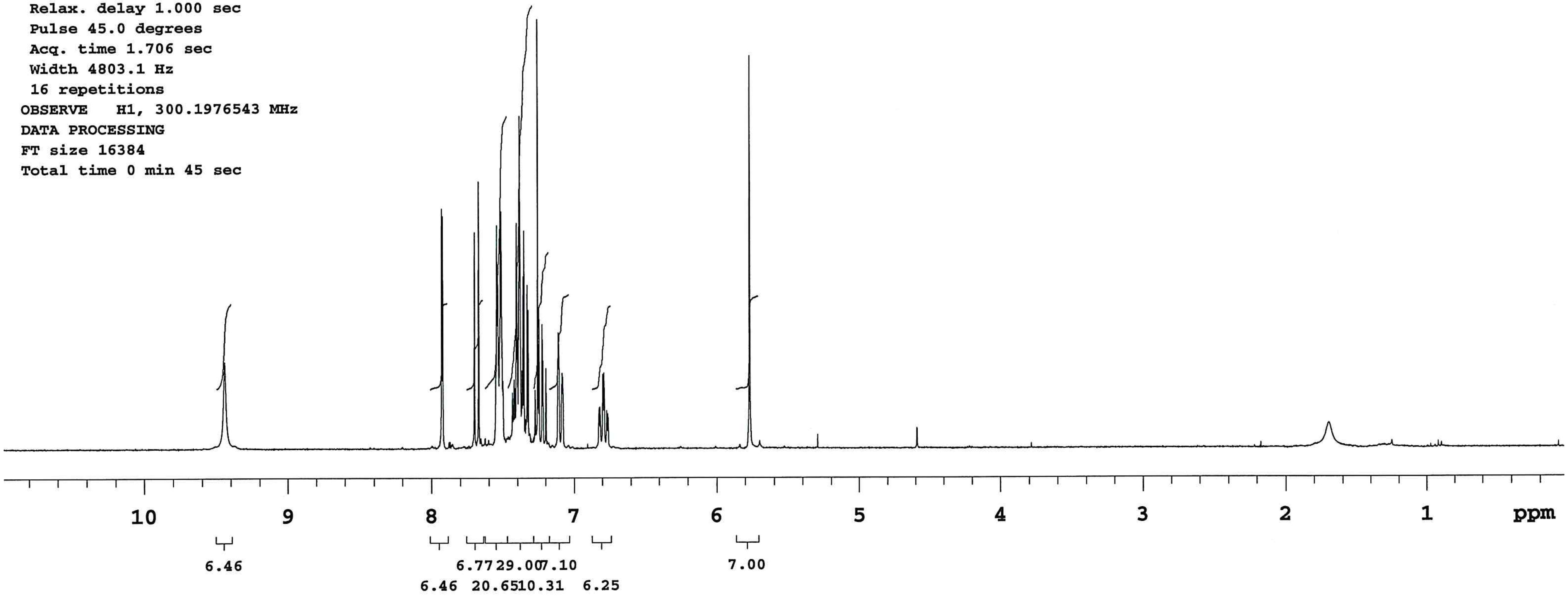

4i

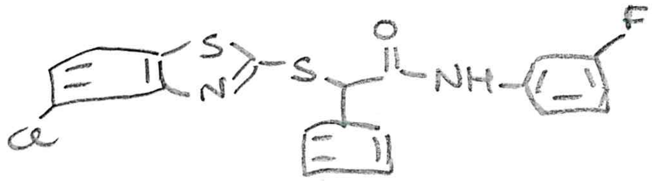

Gradient Shimming

Sample Name:
AA702A
Data Collected on:
m300-mercury300
Archive directory:
/export/home/chempack/vnmrsys/data
Sample directory:

FidFile: CARBON

Pulse Sequence: CARBON (s2pul)
Solvent: cdcl3
Data collected on: Nov 19 2015

Temp. 20.7 C / 293.9 K
Operator: ammazza

Relax. delay 1.000 sec
Pulse 45.0 degrees
Acq. time 0.868 sec
Width 18867.9 Hz
192 repetitions
OBSERVE C13, 75.4847602 MHz
DECOUPLE H1, 300.1991980 MHz
Power 38 dB
continuously on
WALTZ-16 modulated
DATA PROCESSING
Line broadening 0.5 Hz
FT size 32768
Total time 1 hr, 36 min

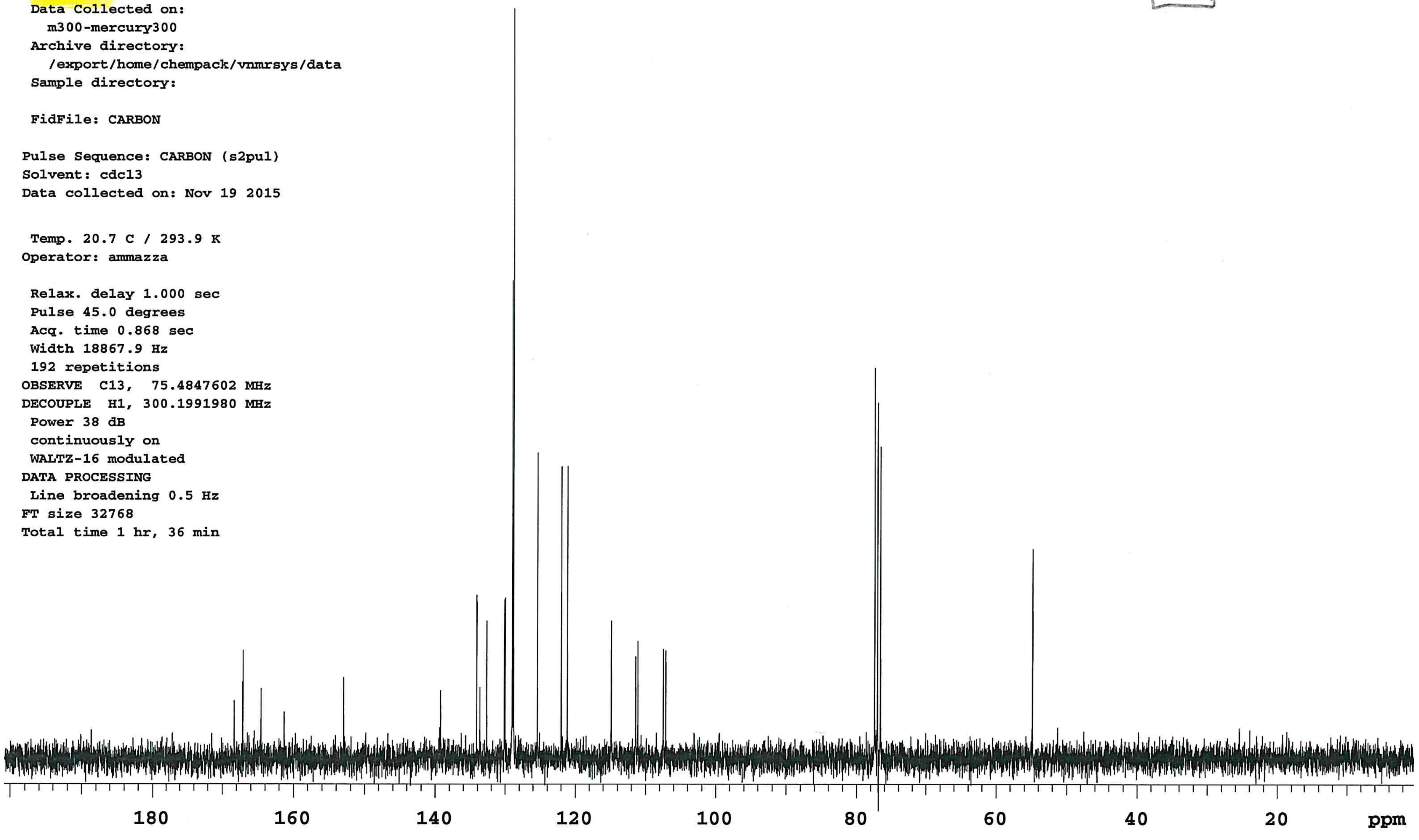

4J

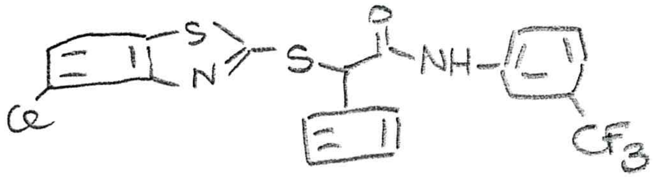

Gradient Shimming

Sample Name:
AA993B
Data Collected on:
m300-mercury300
Archive directory:
/home/ammazza/vnmrsys/data
Sample directory:
MF255_20150422_01
FidFile: AA993B2

Pulse Sequence: PROTON (s2pul)
Solvent: cdcl3
Data collected on: Mar 22 2021

Operator: ammazza

Relax. delay 1.000 sec
Pulse 45.0 degrees
Acq. time 1.706 sec
Width 4803.1 Hz
16 repetitions
OBSERVE H1, 300.1976543 MHz
DATA PROCESSING
FT size 16384
Total time 0 min 45 sec

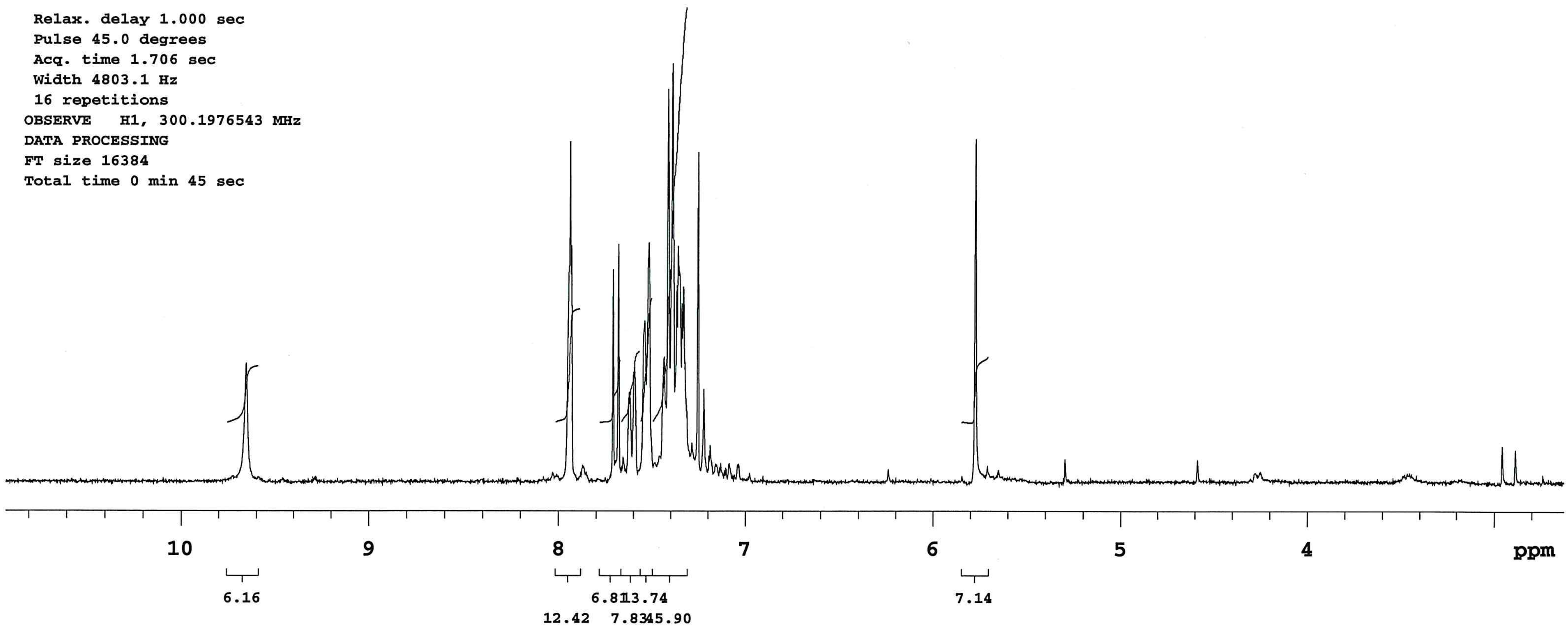

4J

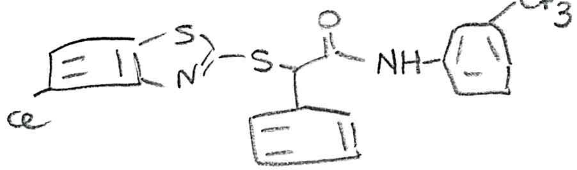

Gradient Shimming

Sample Name:
AA993B
Data Collected on:
m300-mercury300
Archive directory:
/home/ammazza/vnmrsys/data
Sample directory:
AA1008A_20210409_01
FidFile: CARBON

Pulse Sequence: CARBON (s2pul)
Solvent: cdcl3
Data collected on: Apr 15 2021

Temp. 18.2 C / 291.4 K
Operator: ammazza

Relax. delay 1.000 sec
Pulse 45.0 degrees
Acq. time 0.868 sec
Width 18867.9 Hz
31808 repetitions
OBSERVE C13, 75.4847602 MHz
DECOUPLE H1, 300.1991980 MHz
Power 38 dB
continuously on
WALTZ-16 modulated
DATA PROCESSING
Line broadening 0.5 Hz
FT size 32768
Total time 18 hr, 47 min

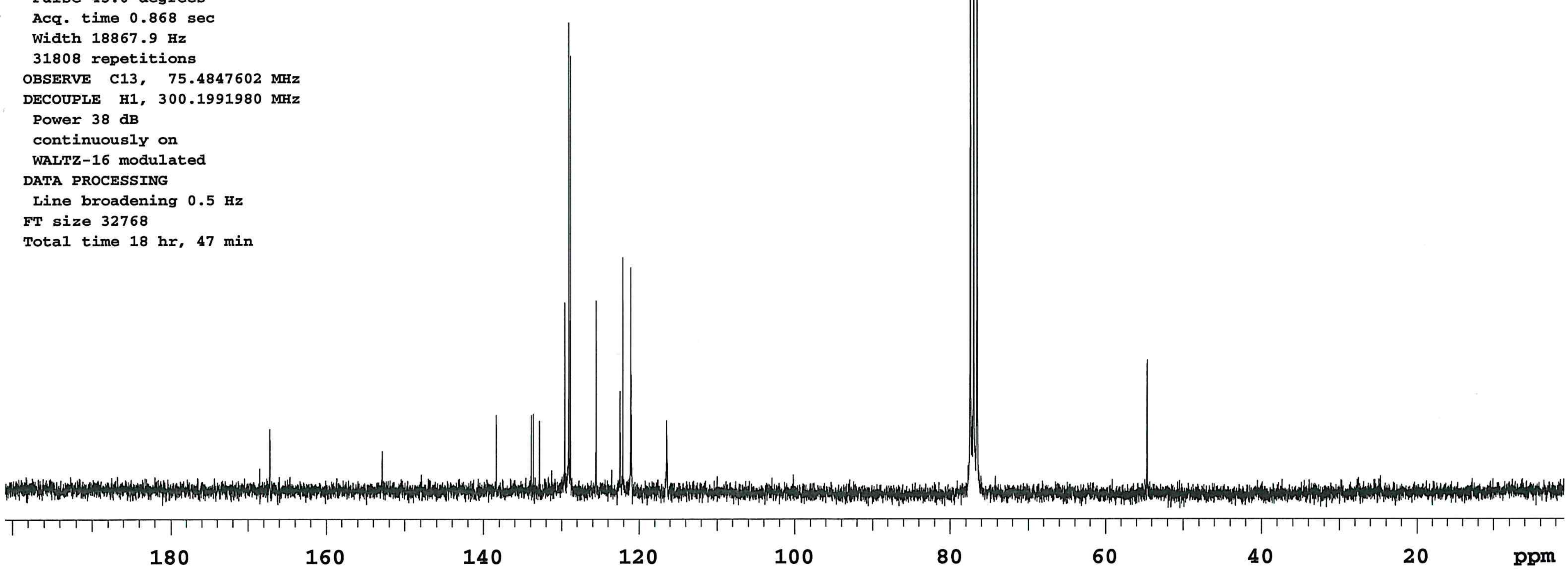

4K

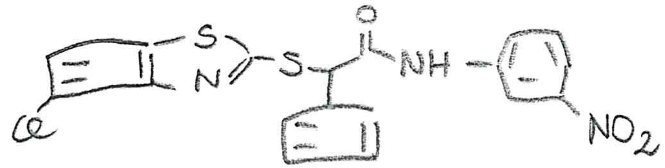

Gradient Shimming

Sample Name:
AA951A
Data Collected on:
m300-mercury300
Archive directory:
/home/ammazza/vnmrsys/data
Sample directory:
MF255_20150422_01
FidFile: AA951A

Pulse Sequence: PROTON (s2pul)
Solvent: cdcl3
Data collected on: Oct 7 2020

Operator: ammazza

Relax. delay 1.000 sec
Pulse 45.0 degrees
Acq. time 1.706 sec
Width 4803.1 Hz
16 repetitions
OBSERVE H1, 300.1976543 MHz
DATA PROCESSING
FT size 16384
Total time 0 min 45 sec

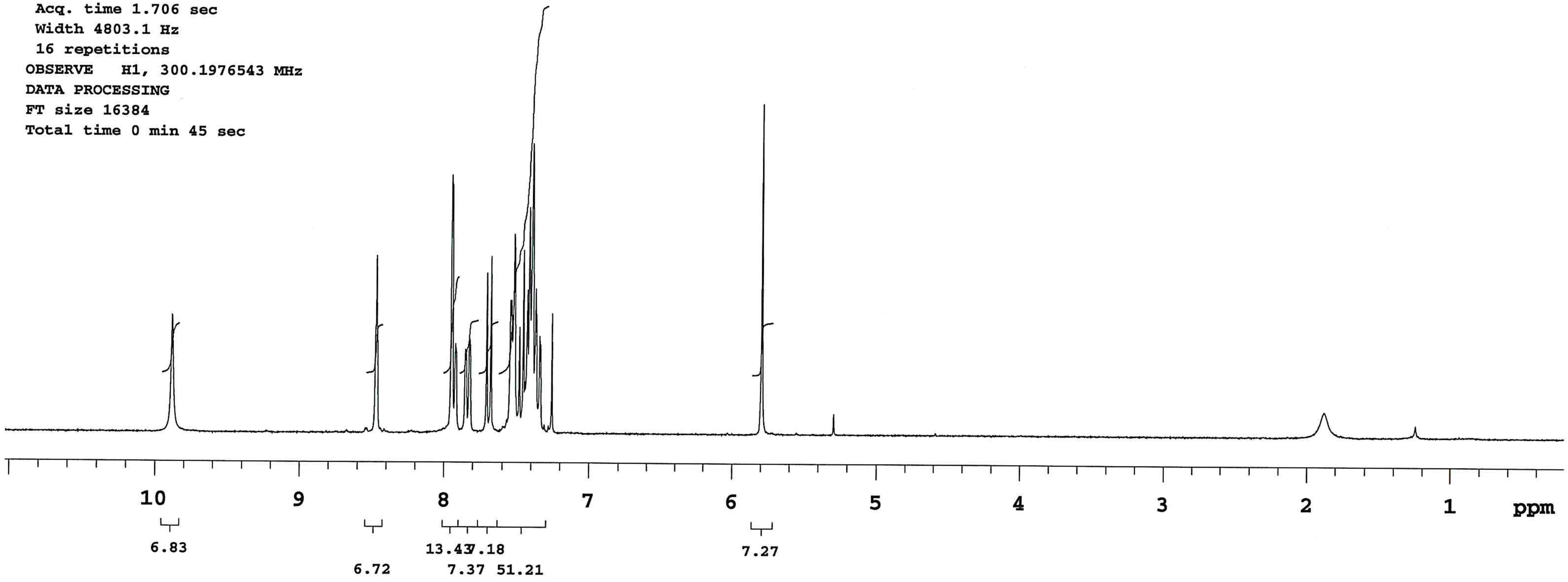

4K

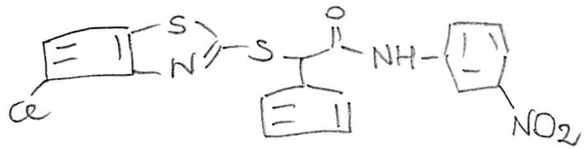

Gradient Shimming

Sample Name:
AA951A
Data Collected on:
m300-mercury300
Archive directory:
/home/ammazza/vnmrsys/data
Sample directory:
MF255_20150422_01
FidFile: CARBON

Pulse Sequence: CARBON (s2pul)
Solvent: cdcl3
Data collected on: Oct 7 2020

Operator: ammazza

Relax. delay 1.000 sec
Pulse 45.0 degrees
Acq. time 0.868 sec
Width 18867.9 Hz
5000 repetitions
OBSERVE C13, 75.4847602 MHz
DECOUPLE H1, 300.1991980 MHz
Power 38 dB
continuously on
WALTZ-16 modulated
DATA PROCESSING
Line broadening 0.5 Hz
FT size 32768
Total time 2 hr, 41 min

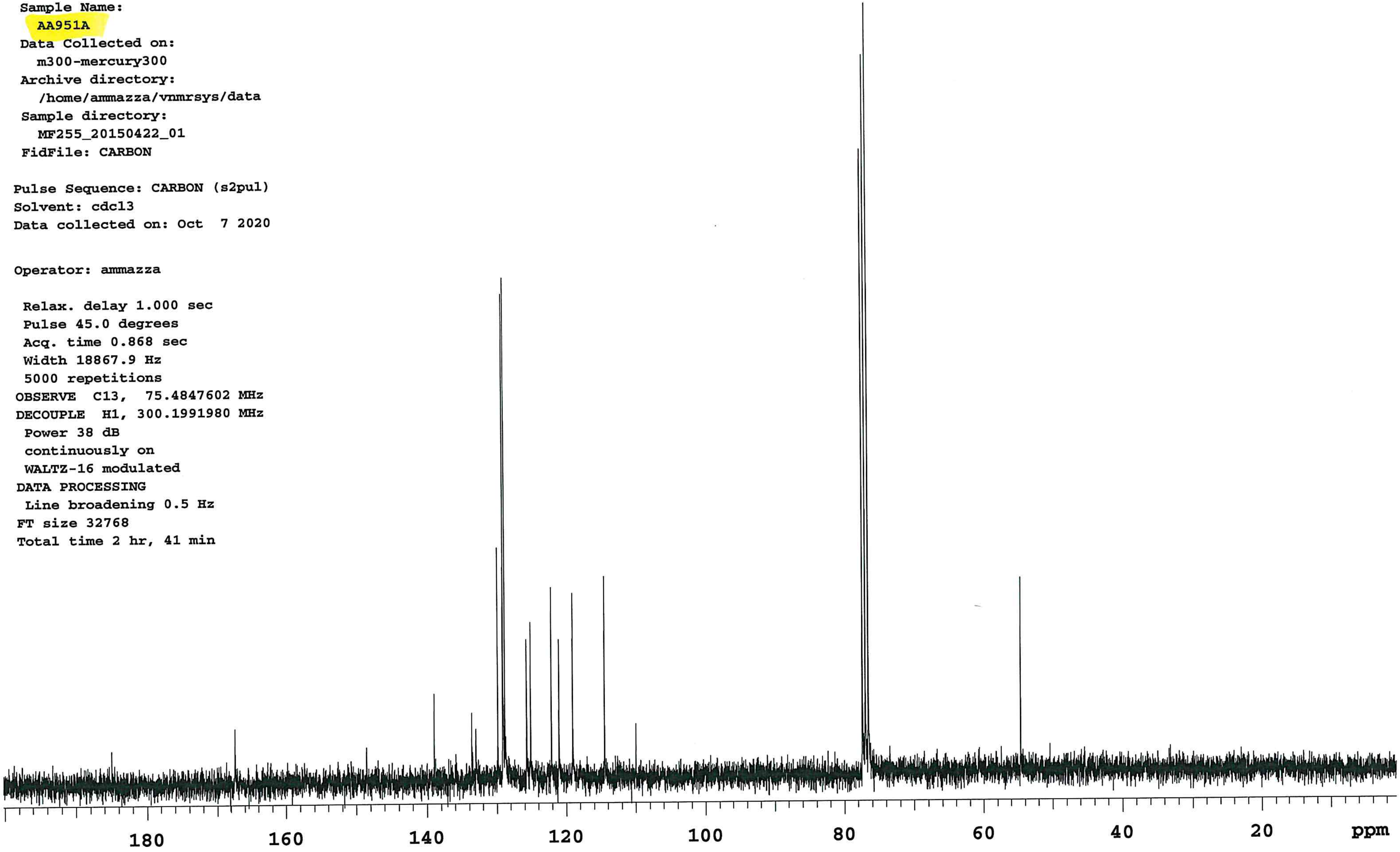

4l

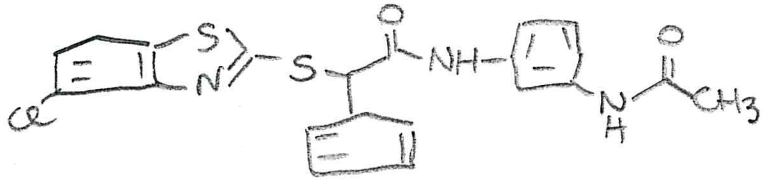

Gradient Shimming

Sample Name:
AA1016ppt
Data Collected on:
m300-mercury300
Archive directory:
/home/ammazza/vnmrsys/data
Sample directory:
AA1008A_20210409_01
FidFile: AA1016ppt

Pulse Sequence: PROTON (s2pul)
Solvent: cdcl3
Data collected on: Apr 22 2021

Operator: ammazza

Relax. delay 1.000 sec
Pulse 45.0 degrees
Acq. time 1.706 sec
Width 4803.1 Hz
16 repetitions
OBSERVE H1, 300.1976543 MHz
DATA PROCESSING
FT size 16384
Total time 0 min 45 sec

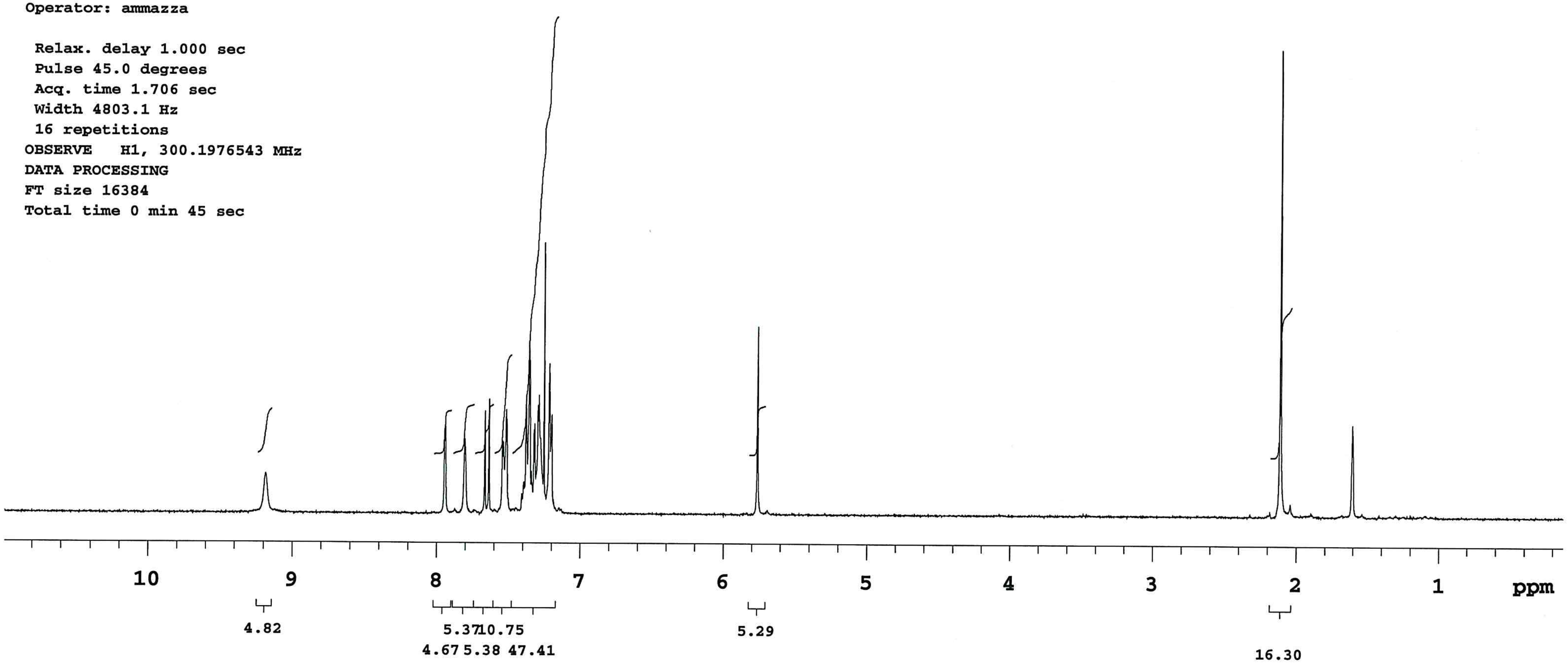

Gradient Shimming

Sample Name:
AA1016ppt
Data Collected on:
m300-mercury300
Archive directory:
/home/ammazza/vnmrsys/data
Sample directory:
AA1008A_20210409_01
FidFile: AA1016ppt_C13

Pulse Sequence: CARBON (s2pul)
Solvent: cdcl3
Data collected on: Apr 26 2021

Operator: ammazza

Relax. delay 1.000 sec
Pulse 45.0 degrees
Acq. time 0.868 sec
Width 18867.9 Hz
1856 repetitions
OBSERVE C13, 75.4847602 MHz
DECOUPLE H1, 300.1991980 MHz
Power 38 dB
continuously on
WALTZ-16 modulated
DATA PROCESSING
Line broadening 0.5 Hz
FT size 32768
Total time 1 hr, 36 min

4l

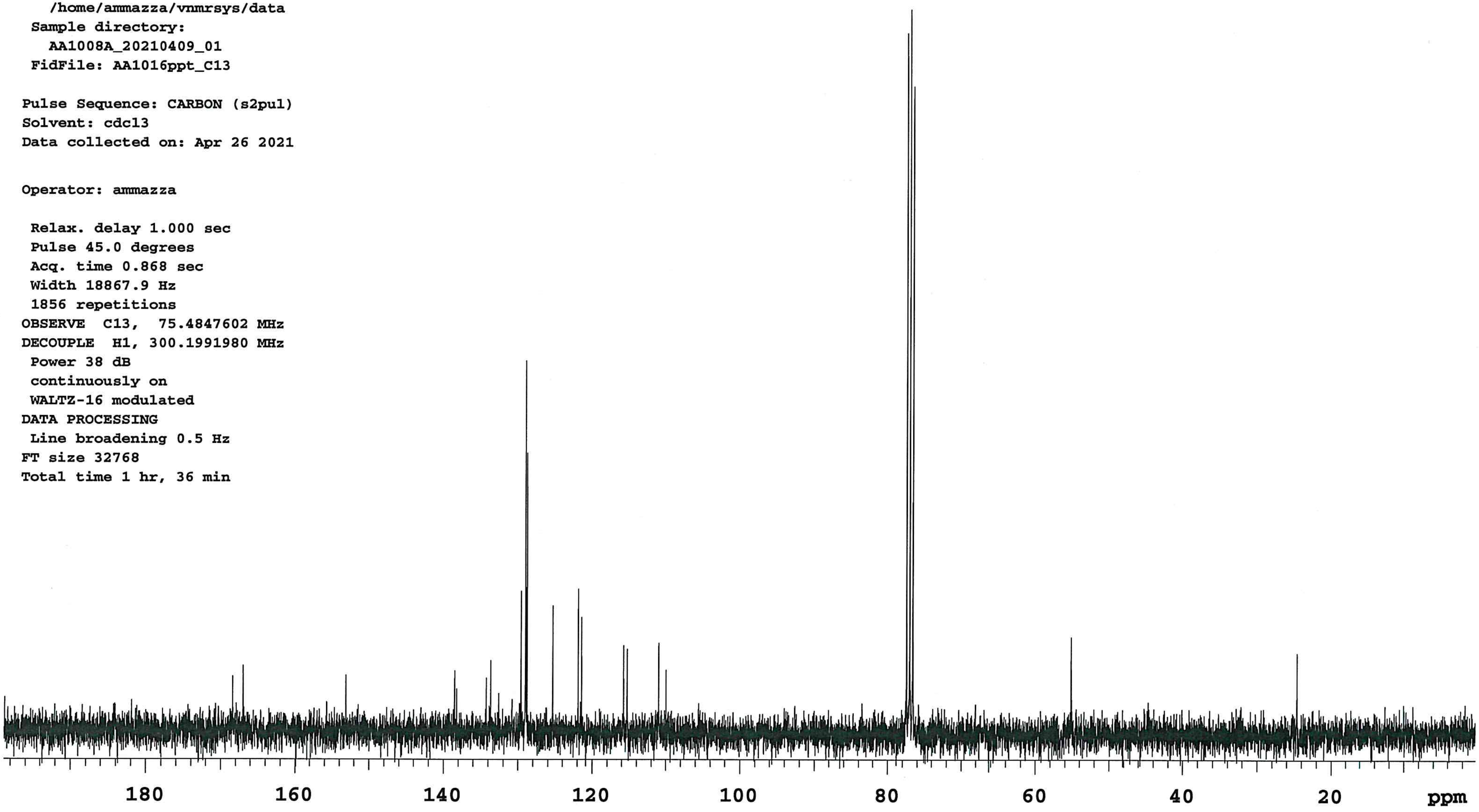

4m

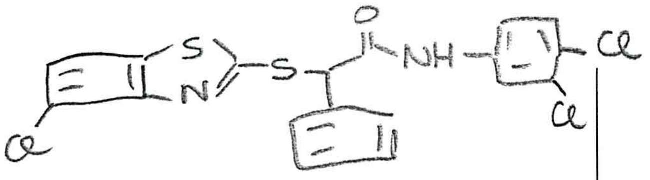

Gradient Shimming

Sample Name:
AA950Admso
Data Collected on:
m300-mercury300
Archive directory:
/home/ammazza/vnmrsys/data
Sample directory:
MF255_20150422_01
FidFile: AA950Admso

Pulse Sequence: PROTON (s2pul)
Solvent: dmso
Data collected on: Oct 14 2020

Operator: ammazza

Relax. delay 1.000 sec
Pulse 45.0 degrees
Acq. time 1.706 sec
Width 4803.1 Hz
16 repetitions
OBSERVE H1, 300.1990802 MHz
DATA PROCESSING
FT size 16384
Total time 0 min 45 sec

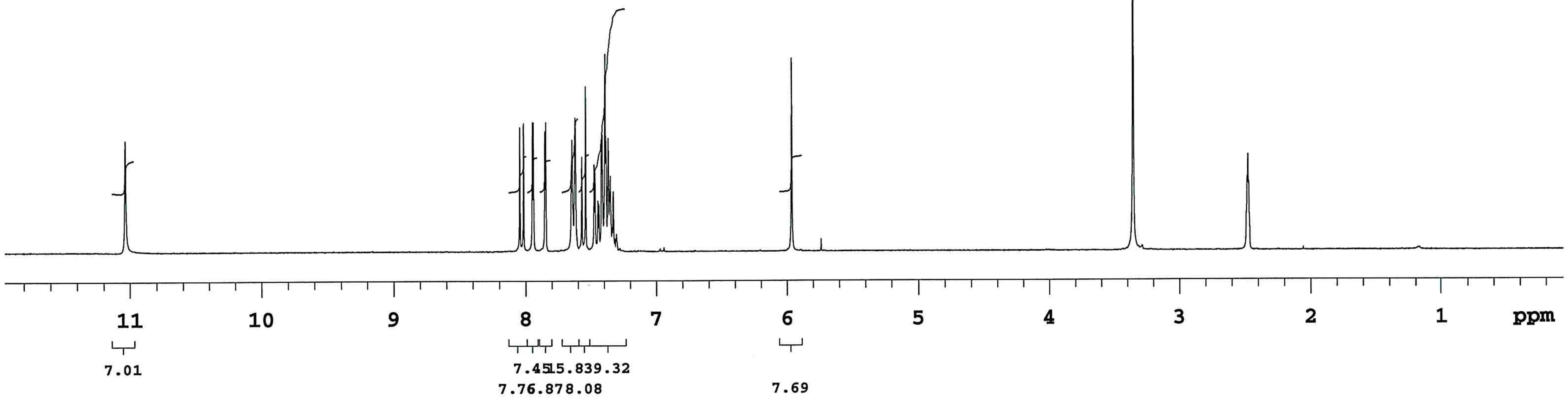

4m

Gradient Shimming

Sample Name:
AA950Admso
Data Collected on:
m300-mercury300
Archive directory:
/home/ammazza/vnmrsys/data
Sample directory:
MF255_20150422_01
FidFile: CARBON

Pulse Sequence: CARBON (s2pul)
Solvent: dmso
Data collected on: Oct 14 2020

Temp. 16.7 C / 289.9 K
Operator: ammazza

Relax. delay 1.000 sec
Pulse 45.0 degrees
Acq. time 0.868 sec
Width 18867.9 Hz
3840 repetitions
OBSERVE C13, 75.4851188 MHz
DECOUPLE H1, 300.2006240 MHz
Power 38 dB
continuously on
WALTZ-16 modulated
DATA PROCESSING
Line broadening 0.5 Hz
FT size 32768
Total time 2 hr, 41 min

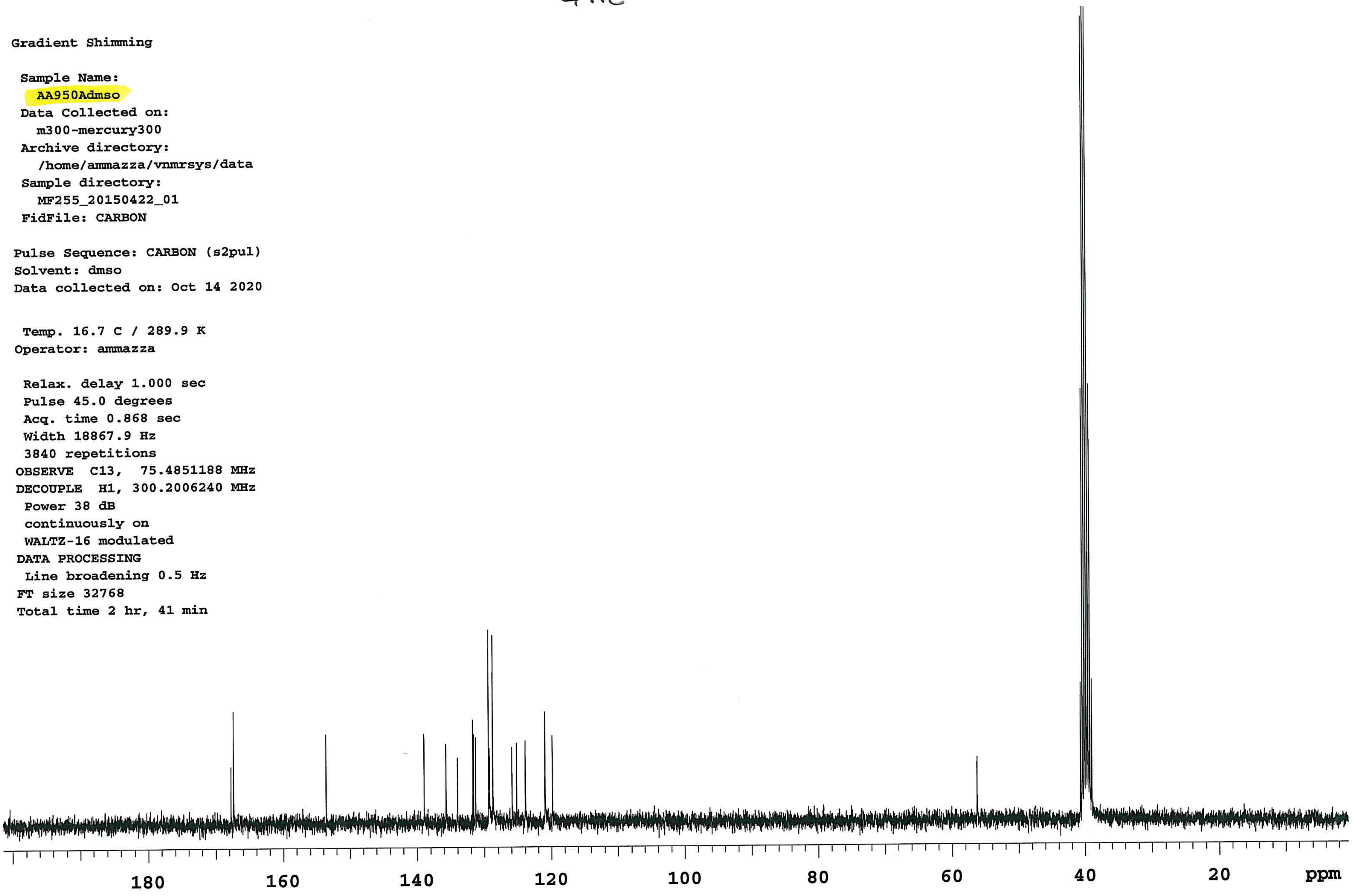

4n

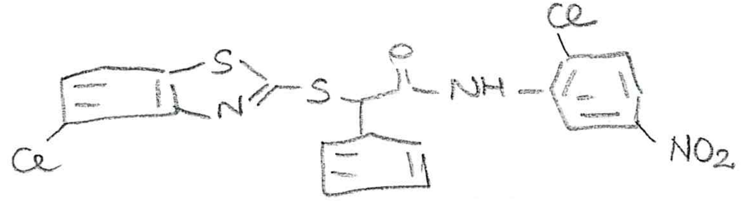

Gradient Shimming

Sample Name:
AA964ppt
Data Collected on:
m300-mercury300
Archive directory:
/home/ammazza/vnmrsys/data
Sample directory:
MF255_20150422_01
FidFile: PROTON

Pulse Sequence: PROTON (s2pul)
Solvent: cdcl3
Data collected on: Feb 12 2021

Operator: ammazza

Relax. delay 1.000 sec
Pulse 45.0 degrees
Acq. time 1.706 sec
Width 4803.1 Hz
16 repetitions
OBSERVE H1, 300.1976543 MHz
DATA PROCESSING
FT size 16384
Total time 0 min 45 sec

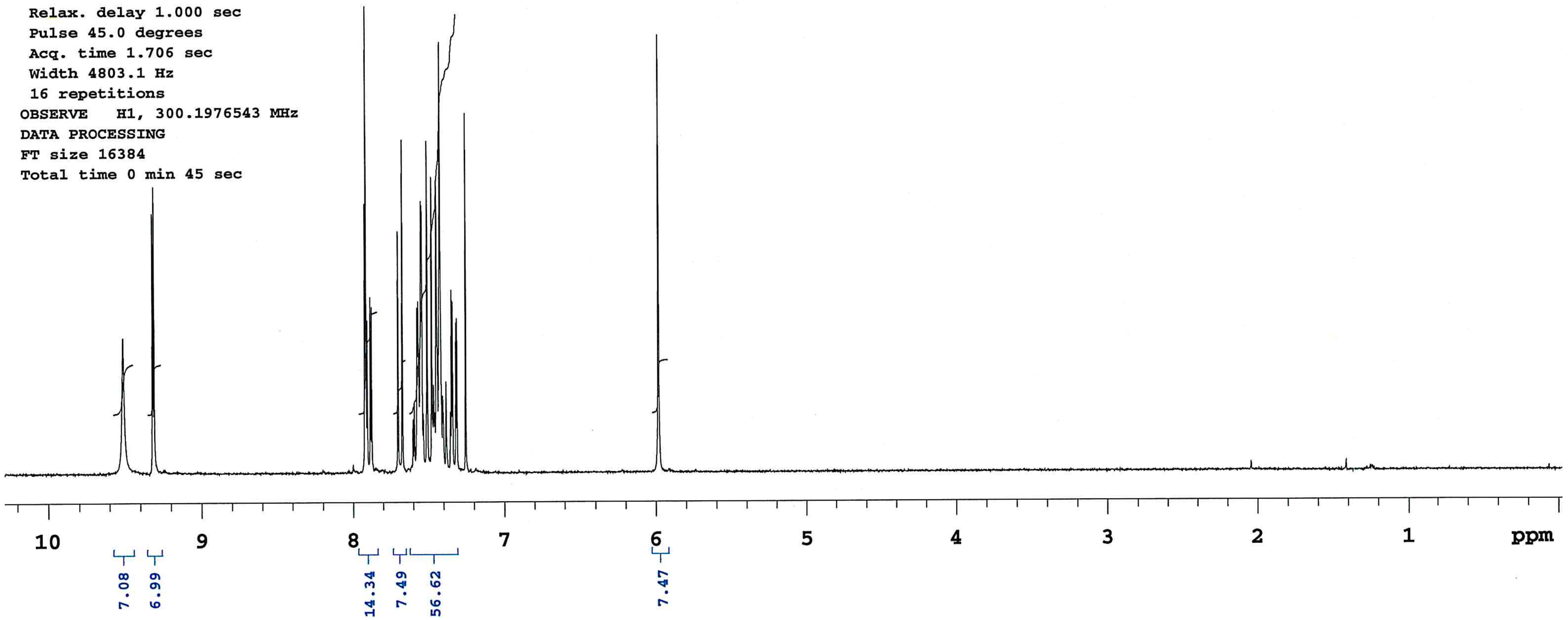

4n

Gradient Shimming

Sample Name:
AA964ppt
Data Collected on:
m300-mercury300
Archive directory:
/home/ammazza/vnmrsys/data
Sample directory:
MF255_20150422_01
FidFile: CARBON

Pulse Sequence: CARBON (s2pul)
Solvent: cdcl3
Data collected on: Feb 12 2021

Temp. 17.6 C / 290.8 K
Operator: ammazza

Relax. delay 1.000 sec
Pulse 45.0 degrees
Acq. time 0.868 sec
Width 18867.9 Hz
1600 repetitions
OBSERVE C13, 75.4847602 MHz
DECOUPLE H1, 300.1991980 MHz
Power 38 dB
continuously on
WALTZ-16 modulated
DATA PROCESSING
Line broadening 0.5 Hz
FT size 32768
Total time 1 hr, 36 min

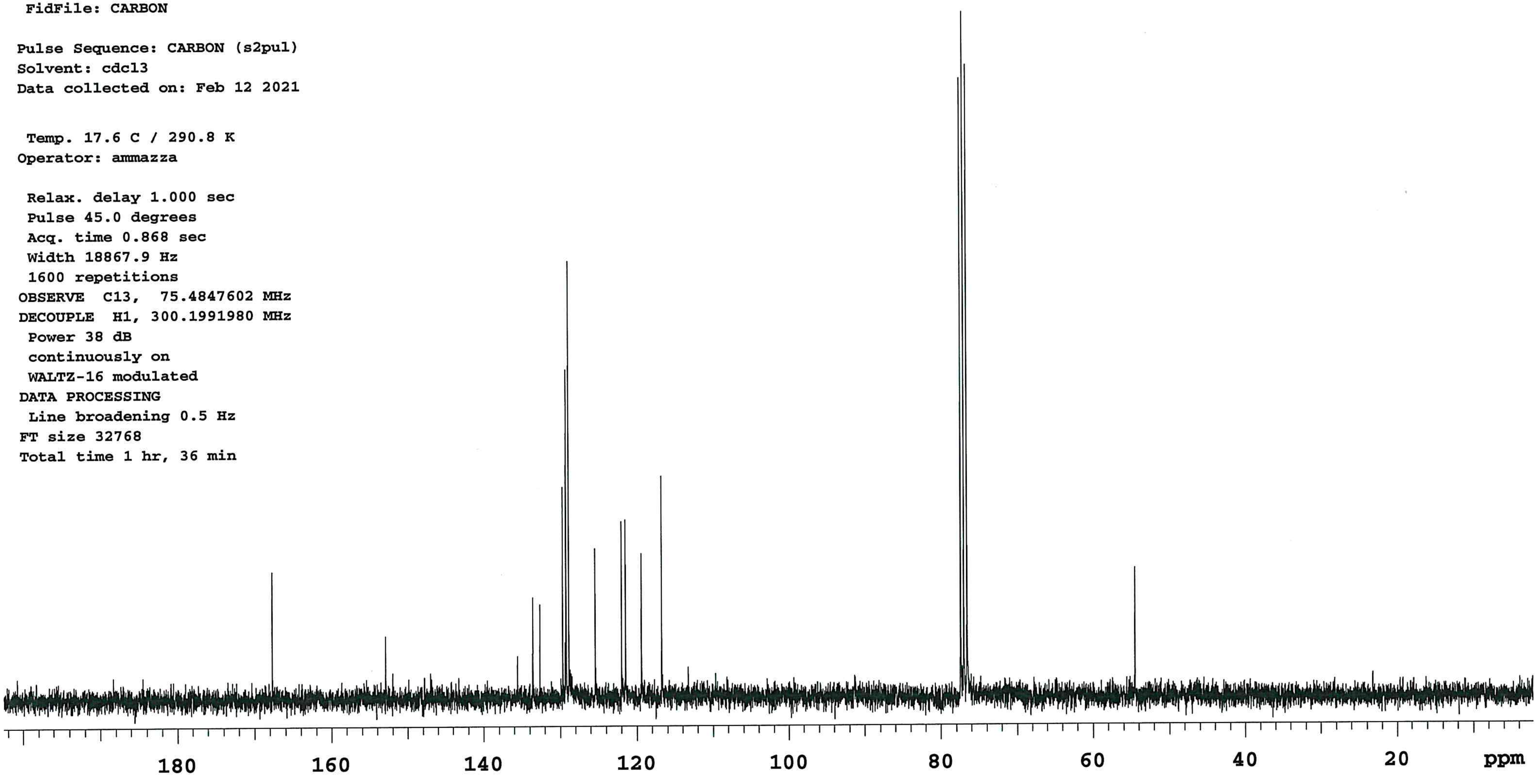

40

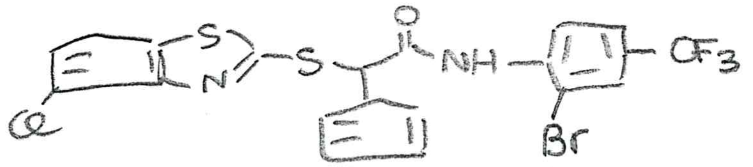

Gradient Shimming

Sample Name:
AA963ppt
Data Collected on:
m300-mercury300
Archive directory:
/home/ammazza/vnmrsys/data
Sample directory:
MF255_20150422_01
FidFile: AA963ppt

Pulse Sequence: PROTON (s2pul)
Solvent: cdcl3
Data collected on: Feb 25 2021

Operator: ammazza

Relax. delay 1.000 sec
Pulse 45.0 degrees
Acq. time 1.706 sec
Width 4803.1 Hz
16 repetitions
OBSERVE H1, 300.1976543 MHz
DATA PROCESSING
FT size 16384
Total time 0 min 45 sec

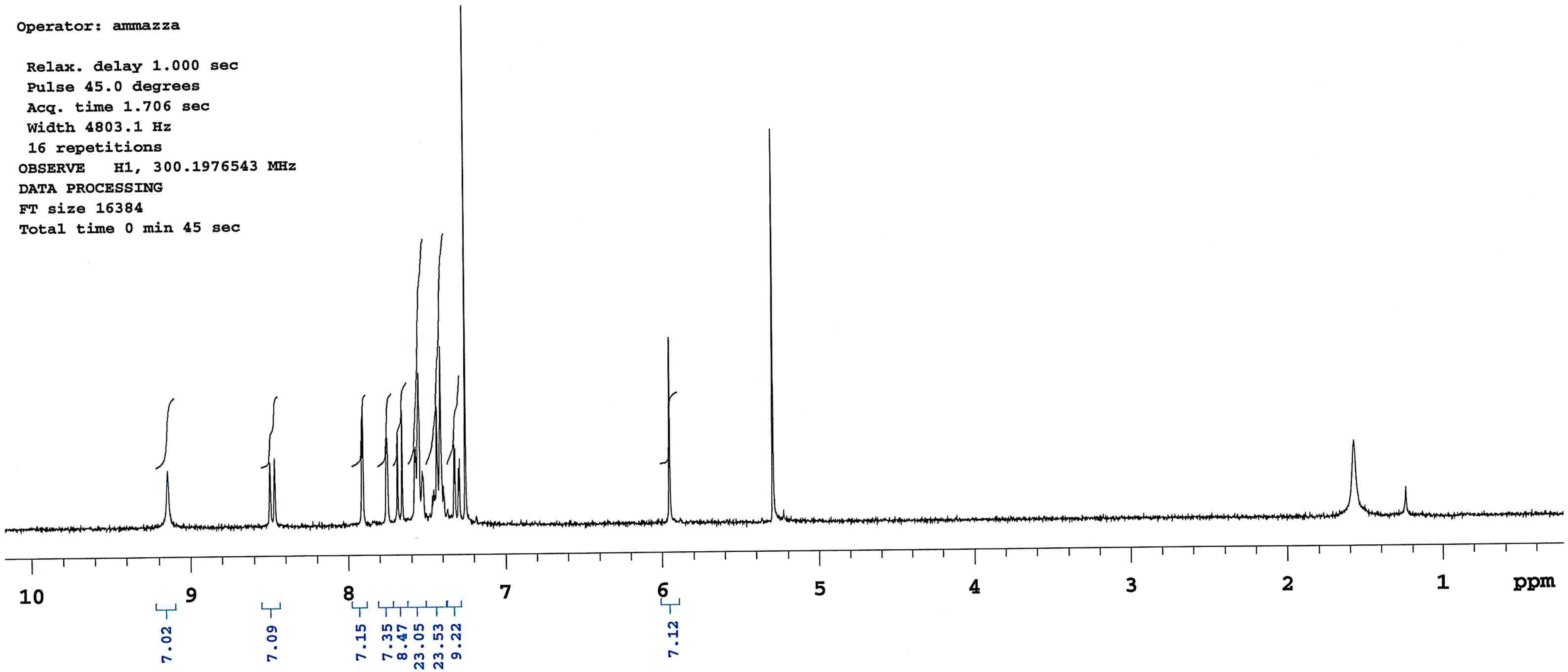

40

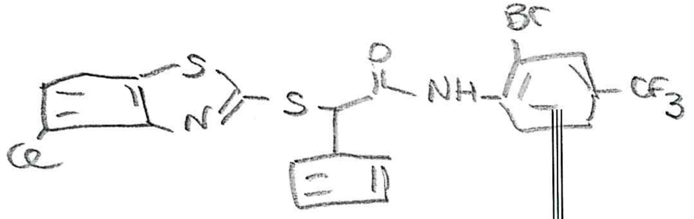

Gradient Shimming

Sample Name:
AA963ppt
Data Collected on:
m300-mercury300
Archive directory:
/home/ammazza/vnmrsys/data
Sample directory:
AA1008A_20210409_01
FidFile: CARBON

Pulse Sequence: CARBON (s2pul)
Solvent: cdcl3
Data collected on: Apr 22 2021

Temp. 17.5 C / 290.6 K
Operator: ammazza

Relax. delay 1.000 sec
Pulse 45.0 degrees
Acq. time 0.868 sec
Width 18867.9 Hz
30976 repetitions
OBSERVE C13, 75.4847602 MHz
DECOUPLE H1, 300.1991980 MHz
Power 38 dB
continuously on
WALTZ-16 modulated
DATA PROCESSING
Line broadening 0.5 Hz
FT size 32768
Total time 53 hr, 41 min

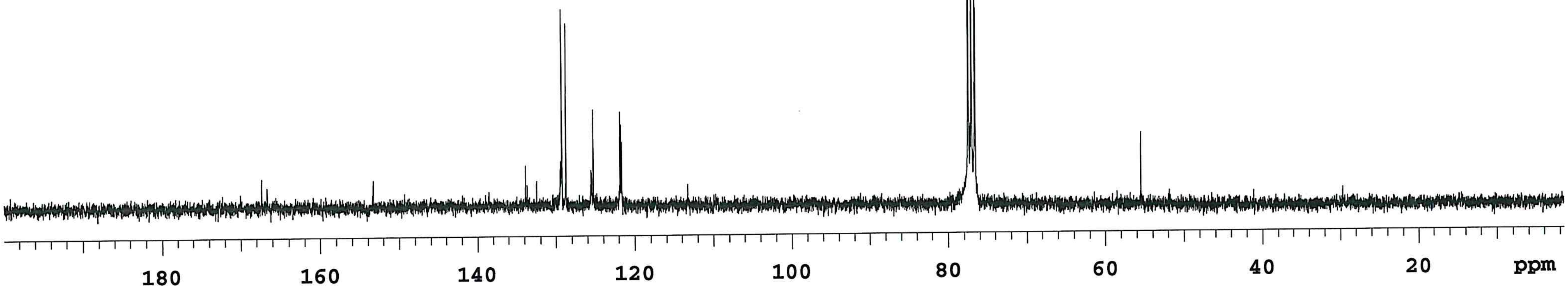

4p

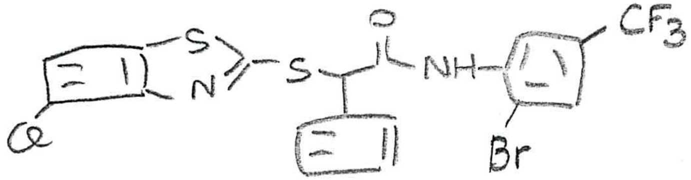

Gradient Shimming

Sample Name:
AA962A
Data Collected on:
m300-mercury300
Archive directory:
/home/ammazza/vnmrsys/data
Sample directory:
MF255_20150422_01
FidFile: AA962Acrist

Pulse Sequence: PROTON (s2pul)
Solvent: cdcl3
Data collected on: Feb 24 2021

Operator: ammazza

Relax. delay 1.000 sec
Pulse 45.0 degrees
Acq. time 1.706 sec
Width 4803.1 Hz
16 repetitions
OBSERVE H1, 300.1976543 MHz
DATA PROCESSING
FT size 16384
Total time 0 min 45 sec

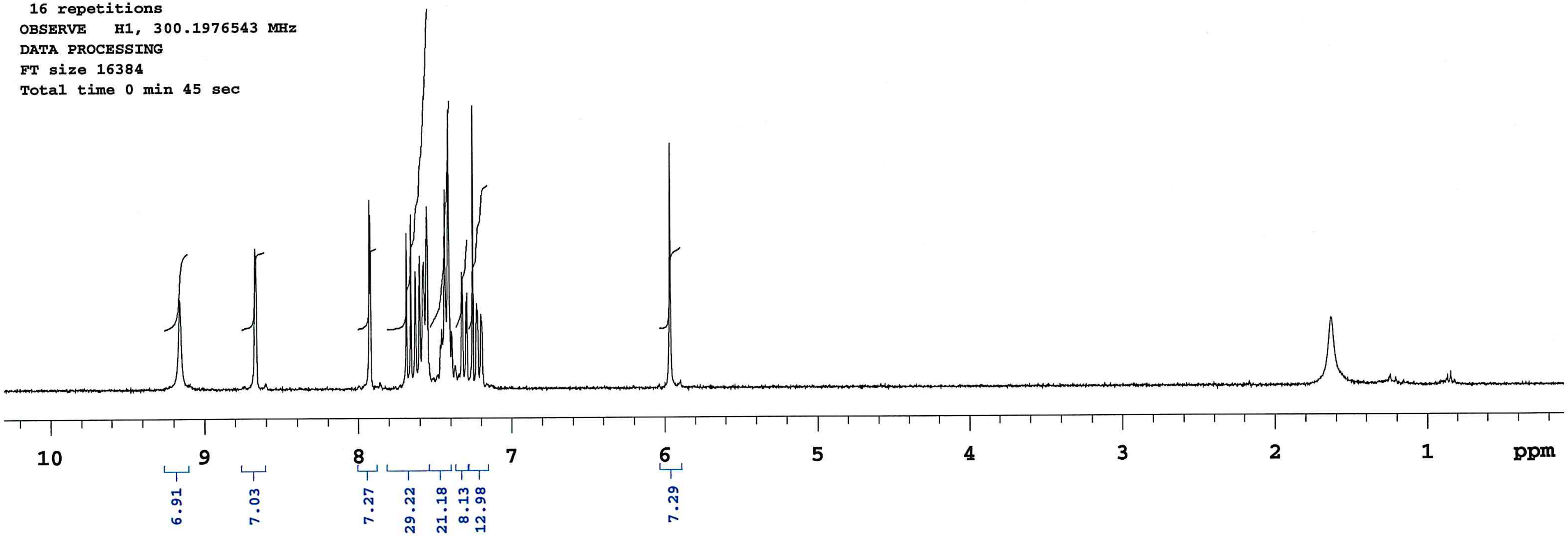

4p

Gradient Shimming

Sample Name:
AA962Acryst.
Data Collected on:
m300-mercury300
Archive directory:
/home/ammazza/vnmrsys/data
Sample directory:
MF255_20150422_01
FidFile: CARBON

Pulse Sequence: CARBON (s2pul)
Solvent: cdcl3
Data collected on: Mar 3 2021

Temp. 17.4 C / 290.6 K
Operator: ammazza

Relax. delay 1.000 sec
Pulse 45.0 degrees
Acq. time 0.868 sec
Width 18867.9 Hz
2624 repetitions
OBSERVE C13, 75.4847602 MHz
DECOUPLE H1, 300.1991980 MHz
Power 38 dB
continuously on
WALTZ-16 modulated
DATA PROCESSING
Line broadening 0.5 Hz
FT size 32768
Total time 2 hr, 8 min

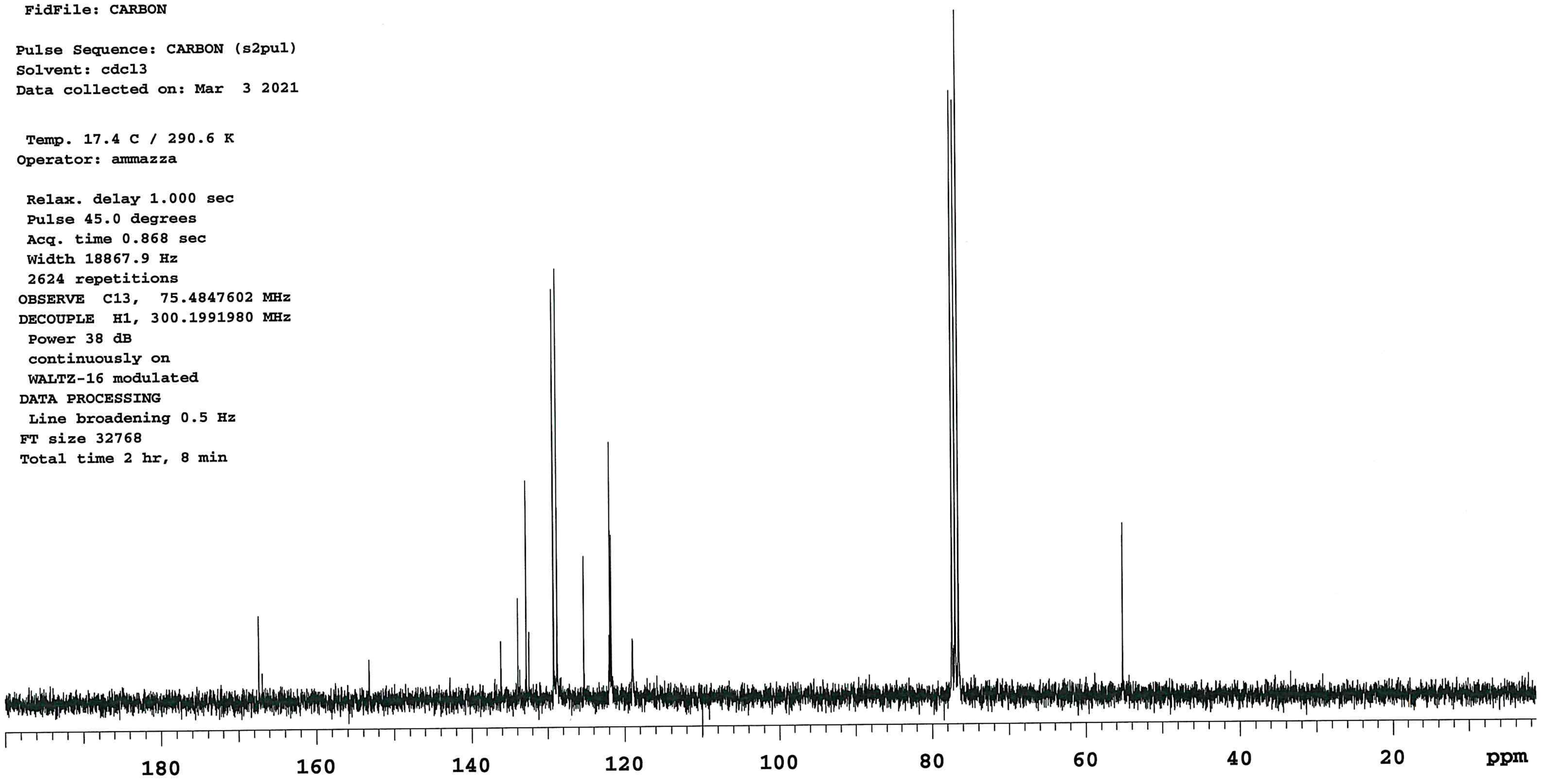

Supplement: Supplementary file 1 [file pharmaceuticals-15-00937-s001.zip › pharmaceuticals-1802529-supplementary.pdf]
